# Establishment, Implementation, and Impacts of the Observatory on Student Mental Health in Higher Education in Quebec, Canada: Protocol for a Mixed Methods Research Program

**DOI:** 10.2196/83225

**Published:** 2026-04-22

**Authors:** Julie Lane, Benjamin Gallais, François Lauzier-Jobin, Sèverine Lanoue, Félix Guay-Dufour, Rémi Paré-Beauchemin, Rachel Guertin, Christiane Bergeron-Leclerc, Saliha Ziam, Marie-Claude Lallier Beaudoin, Martine Shareck, Véronique Boudreault, Marie-Ève Langelier, Steve Geoffrion, Nancy Beauregard, Bianca B-Lamoureux, Magaly Brodeur, Esther McSween-Cadieux, Audrey Dupuis, Jonathan Smith, Isabelle Thibault, Noémie Beauregard

**Affiliations:** 1 Department of School and Social Adaptation Studies, University of Sherbrooke, 2500 University Boulevard, Sherbrooke, QC, J1N 3C6, Canada, 1 819 993-2306; 2 Department of Health Sciences, University of Quebec at Chicoutimi, Chicoutimi, QC, Canada; 3 School of Nursing Science, University of Sherbrooke, Sherbrooke, QC, Canada; 4 University of Sherbrooke, Sherbrooke, QC, Canada; 5 Faculty of Education, University of Sherbrooke, Sherbrooke, QC, Canada; 6 Department of Humanities and Social Sciences, University of Quebec at Chicoutimi, Chicoutimi, QC, Canada; 7 School of Business Administration, TELUQ University, Montreal, QC, Canada; 8 Department of Psychology, University of Sherbrooke, Sherbrooke, QC, Canada; 9 Faculty of Medicine and Health Sciences, University of Sherbrooke, Sherbrooke, QC, Canada; 10 Faculty of Physical Activity Sciences, University of Sherbrooke, Sherbrooke, QC, Canada; 11 School of Psychoeducation, University of Montreal, Montreal, QC, Canada; 12 School of Industrial Relations, University of Montreal, Montreal, QC, Canada; 13 Department of Education, TELUQ University, Montreal, QC, Canada; 14 Department of Family Medicine and Emergency Medicine, University of Sherbrooke, Sherbrooke, QC, Canada; 15 Faculty of Education, University of Moncton, Moncton, NB, Canada; 16 Department of Preschool and Primary Education, University of Sherbrooke, Sherbrooke, QC, Canada; 17 Department of Psychoeducation, University of Sherbrooke, Sherbrooke, QC, Canada

**Keywords:** observatory, higher education, mental health, impacts, students, knowledge mobilization

## Abstract

**Background:**

Research is needed to better understand mental health (MH) problems among higher education (HE) students and how to address them. The Observatory on Student Mental Health in Higher Education (OSMHHE) brings together 350 members across Quebec (Canada) and internationally. Its mission is to develop, promote, and disseminate knowledge to foster a culture that supports student MH in HE.

**Objective:**

This study aims to describe the OSMHHE’s research protocol, which consists of three objectives: (1) establishing a portrait of students’ MH and its determinants, (2) identifying and evaluating a variety of MH practices, and (3) assessing the implementation and impacts of the OSMHHE as a knowledge mobilization infrastructure.

**Methods:**

Objective 1 will be achieved through a provincial survey using a cross-sectional, repeated-measures design with 2 data collections (November 2024 and 2026) targeting the entire Quebec HE student population. Dimensions, indicators, and scales were selected based on conceptual frameworks, a systematic literature review, and Delphi methods. Analyses will include descriptive statistics by education levels; inferential analyses comparing subpopulations; multiple regressions, logistic models, and linear mixed models to identify MH determinants; and repeated-measures ANOVA to examine temporal changes. Objective 2 will evaluate the implementation, sustainability, scale-up, and impacts of MH practices using mixed methods. Analyses may include descriptive and comparative statistics, correlations, structural equations modeling (path analysis), and qualitative content or thematic analyses. Objective 3 will draw on the framework of Ziam et al to assess knowledge mobilization strategies. A developmental evaluation approach and convergent mixed methods design within a case study will be used to assess the OSMHHE’s implementation and impacts. Qualitative data will include semistructured individual and group interviews with OSMHHE members, addressing topics such as roles, decision-making processes, facilitators and barriers, and outcomes. Additional qualitative sources will include diverse documents (eg, meeting agendas, reports). Quantitative data will come from questionnaires completed by members examining levels of engagement and satisfaction, challenges and barriers, and impacts of the OSMHHE’s activities and knowledge mobilization practices. Qualitative data will be analyzed using content analyses. Quantitative data will be examined using descriptive, comparative, and correlational analyses.

**Results:**

This project is funded from February 2023 to February 2028. The first provincial survey took place in November 2024, collecting data from 32,212 students in 77 HE institutions. Analyses are underway, and a first report was released in November 2025. Approximately 20 student MH practices are currently being evaluated.

**Conclusions:**

The OSMHHE provincial survey will provide portraits of students’ MH in HE in Quebec and its determinants to better guide MH practices and institutional decision-making. Evaluating MH practices will advance knowledge of their effectiveness. Assessing the implementation of the OSMHHE will help deepen our understanding of knowledge mobilization infrastructures designed to support student MH in HE.

## Introduction

### Background

Higher education institutions (HEIs) are internationally recognized as environments that support the mental health (MH) of their communities [1,2]. This is in line with the Healthy Universities and Health Promoting Universities movement [3] and the Okanagan Charter, to which 45 countries adhere [4]. Despite this recognition, several studies have highlighted the precarious MH of students in higher education (HE) [5-8]. A study involving 14,348 students from 19 universities in 8 countries showed that 38.4% of respondents had experienced at least 1 MH problem (eg, anxiety disorder, depressive disorder, substance use disorder) over the course of their studies [8]. These MH problems can impair students’ cognitive function, psychosocial adjustment, motivation, and academic performance, while being associated with reduced well-being, lower social participation and academic satisfaction, as well as increased risks of dropping out of school and of suicide [9-14].

In Quebec (Canada), a 2020 survey of 1815 college students found that 33.7% had clinical symptoms consistent with a depressive disorder and that 24.9% had clinical symptoms of generalized anxiety disorder [15]. A province-wide survey carried out by the Quebec Student Union found that 58% of university students were experiencing a high level of psychological distress and that 1 in 5 had depressive symptoms severe enough to warrant medical or psychological support [16]. Another survey by the College Students Federation of Quebec reported that 63.8% of students felt their psychological health had deteriorated during the pandemic [17]. Finally, a Quebec Student Union survey also conducted during the pandemic showed that 51% of the student community felt that their level of distress had increased during this period [18].

Although the surveys presented above highlight MH issues among students in HE, further research is needed to deepen knowledge, monitor changes over time, and reduce certain methodological biases. Indeed, many surveys rely on recruitment strategies (eg, via social networks) that may limit sample representativeness [19,20]. Moreover, some surveys use nonstandardized instruments, use methodologies that vary across institutions, or rely on cross-sectional designs. These limitations make it difficult to develop a reliable province-wide portrait of student MH, identify its risk and protective factors, conduct longitudinal follow-up, and formulate actionable recommendations. Finally, few studies adopt a holistic approach that considers both the multiple dimensions of student MH and the systemic factors or determinants that influence it, whether individual (eg, identity, psychosocial skills), social (eg, social support, discrimination), or environmental (eg, sense of belonging to the university, campus climate).

In response to MH issues, HEIs report difficulties coping with increased demand for consultations and the growing severity of problems encountered [21-23]. A meta-analysis has shown that traditional psychological services struggle to meet the diverse needs of students [24]. Moreover, these services are often fragmented, tend to respond to needs in a reactive and ad hoc manner, are underfunded, and, consequently, require substantial additional resources [25].

On the other hand, it remains difficult to ascertain which practices are most effective or promising for supporting students’ MH in HE [26-32]. In Quebec, as elsewhere, little information is available on the implementation and evaluation of these interventions [33,34]. Despite these limitations, several meta-analyses have concluded that the interventions assessed (eg, mindfulness meditation) are effective, showing small-to-moderate effect sizes on symptoms of stress, anxiety, and depression [26-29,31,32,35,36]. However, Huang et al [30] note that interventions targeting the general student population do not demonstrate significant efficacy for more severe MH problems. This finding is consistent with another meta-analysis reporting that, among students reporting high levels of stress, more structured and longer-term intervention programs are particularly effective [35]. Finally, several stakeholders in the field have observed a proliferation of practices and innovations being implemented in HEIs without undergoing any evaluation [37].

Initiatives to improve students’ MH require a broad cultural transformation within academic environments, spanning from the promotion to implementation of practices to foster MH [38-41]. Producing research results, and even disseminating, synthesizing, or contextualizing them within HEIs to promote their use, is not sufficient. There is also a need to support the adaptation of practices and the development of both individual and collective leadership related to MH. HEIs would benefit from receiving support to promote, implement, evaluate, sustain, and scale up MH practices across multiple institutions [42]. A systematic review on the sustainability of public health interventions implemented in educational settings identified key enabling conditions (eg, support from leaders, coconstruction approaches) and contextual barriers (eg, insufficient human and financial resources) that can promote or hinder their implementation [43]. However, to date, little is known about the implementation of promising practices to promote MH in educational settings, or about the factors that influence their adoption and sustainability [44,45]. In light of this, supporting knowledge mobilization (KM) to promote and maintain a culture conducive to student MH in HE is clearly relevant.

### Objective of the Study

To overcome these limitations, the Observatory on Student Mental Health in Higher Education (OSMHHE) (*Observatoire sur la santé mentale étudiante en enseignement supérieur*), funded by the Fonds de recherche du Québec (FRQ, 2023‐2027), aims to contribute to the advancement of knowledge and the KM development and practice in order to promote and maintain a culture conducive to enhancing student MH in HE [46]. This study describes the OSMHHE research protocol, as submitted to the FRQ, which is akin to a research program. The study presents the OSMHHE logic model and its components, the objectives of the research protocol, the research methodology, the anticipated results, and a discussion.

### OSMHHE Logic Model

#### Overview

**Figure 1.**
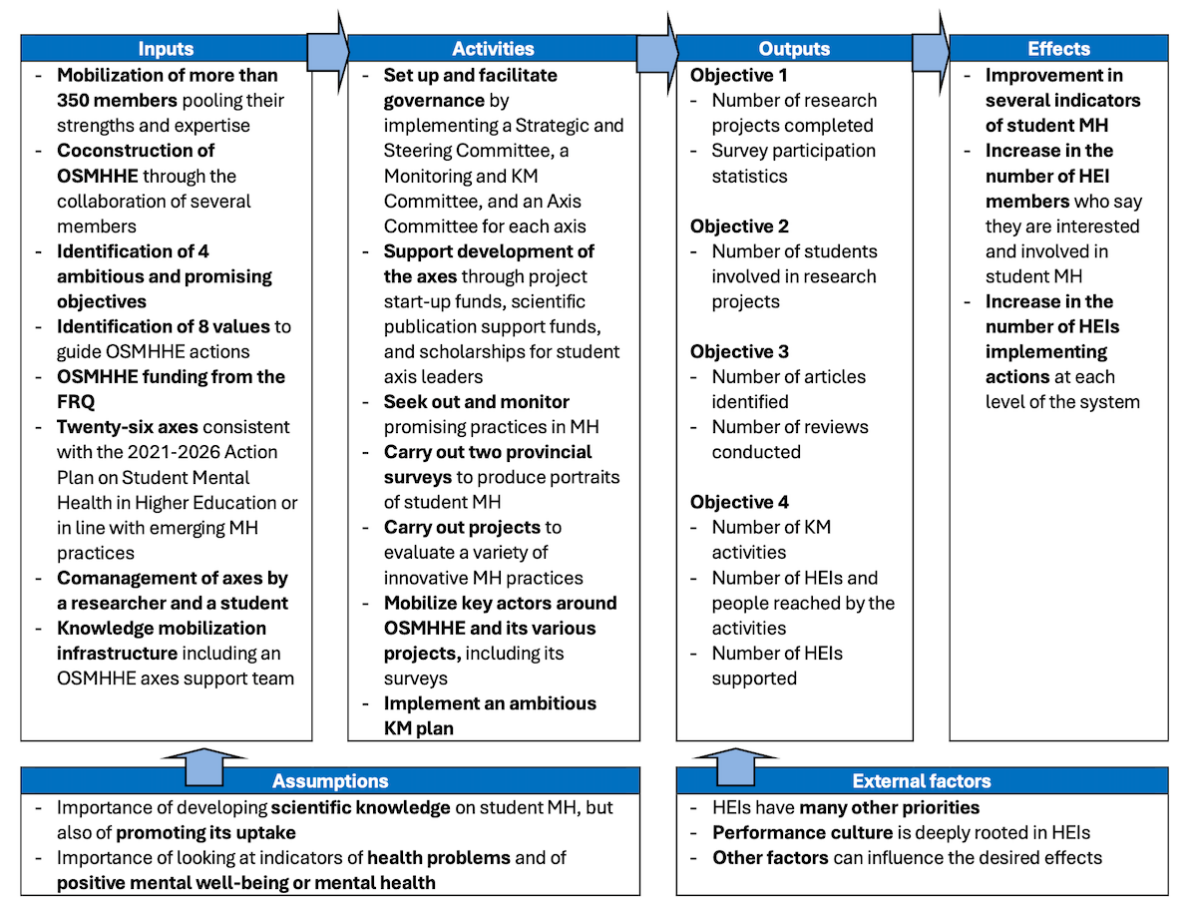
Observatory on Student Mental Health in Higher Education (OSMHHE) logic model. FRQ: Fonds de recherche du Québec; HEI: higher education institution; KM: knowledge mobilization; MH: mental health.

Drawing on the Quebec government’s 2021‐2026 Action Plan on Student Mental Health in Higher Education (*Plan d’action sur la santé mentale étudiante en enseignement supérieur*) [47], the OSMHHE aims to (1) develop scientific knowledge through research projects and a survey on student MH and promising MH practices. Since there are few evidence-based practices in the field of student MH in HE, we use the concept of promising practice. A promising practice must have demonstrated a minimal effect (eg, observed positive changes toward goal attainment), evidence of correct quality (eg, in the early stages of evaluation), or high potential to produce similar positive results in other contexts [48]. In addition, the concept of practice encompasses initiatives, programs, measures, services, and so forth. Furthermore, the OSMHHE aims to (2) contribute to training and mobilizing students with regard to MH in order to create positive leaders; (3) seek out and monitor promising practices in MH promotion, awareness, prevention, and intervention; and (4) promote KM by implementing activities to support changes in culture and practices. The OSMHHE logic model is illustrated in Figure 1, and more information can be found on the OSMHEE website [46].

#### OSMHHE Axes and Governance

The OSMHHE brings together more than 350 professors, researchers, students, and collaborators, as well as a number of cross-sectoral partners (eg, government bodies, HEIs, community organizations). Eight values guide their actions and projects: (1) democratization of access to knowledge; (2) nonhierarchization of knowledge and human relationships; (3) benevolence and authenticity; (4) collaboration; (5) equity, diversity, and inclusion; (6) commitment and determination; (7) creativity; and (8) excellence.

**Figure 2.**
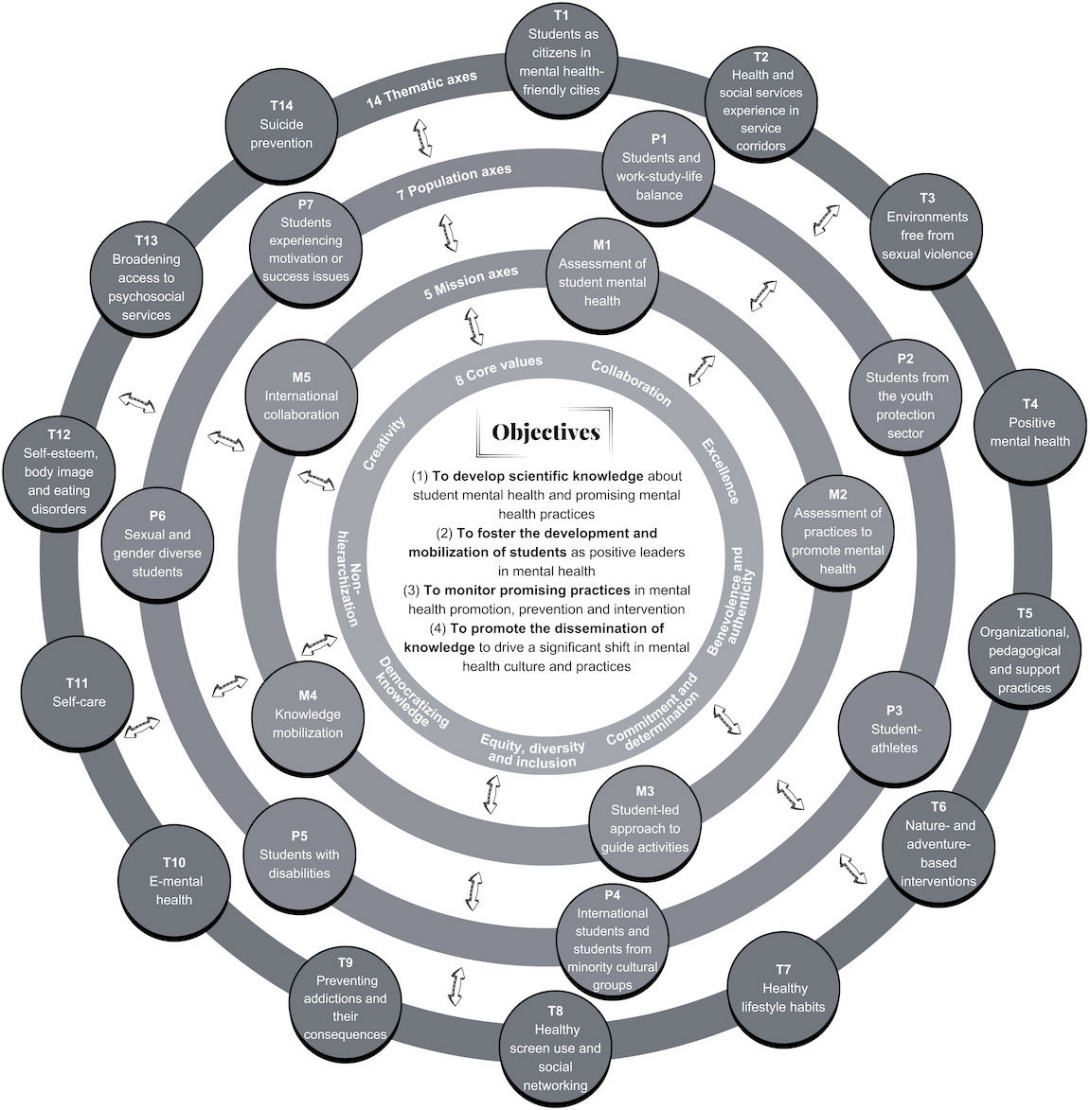
The 26 mission, population, and thematic axes of the Observatory on Student Mental Health in Higher Education (OSMHHE).

These members collaborate around 26 mission, population, and theme axes (Figure 2). Each axis is supported by a coinvestigator and a student coleader and brings together a variety of members within an axis committee. The OSMHHE has also established a strategic committee and a monitoring and KM committee.

#### The Four Pillars of OSMHHE

##### Overview

To achieve its objectives, the OSMHHE is grounded on four pillars: (1) adopting a holistic conception of student MH, (2) fostering a participatory “by-and-for students” approach, (3) implementing governance based on the Teal organizational model [49], and (4) defining itself as a knowledge mobilization infrastructure (KMI) to support change in culture and practices in HEIs. These pillars make the OSMHHE a unique infrastructure that stands out from others, such as Canada’s Student Mental Health Network [50,51] and the Community Engagement Course and Action Network [52]. Indeed, these networks are more oriented toward KM, whereas the OSMHHE combines a dual mission of advancing knowledge through both research and KM.

##### A Holistic Conception of Student MH and Its Determinants

The OSMHHE adopts a holistic conception of MH, defined as “a state of well-being in which the individual realizes his or her own abilities, can cope with the normal stresses of life, can work productively and fruitfully, and is able to make a contribution to his or her community” (p. 1) [53]. It therefore does not consist solely of the absence of disease [53]. Keyes’ [54] complete state model of MH proposes the combination of a first continuum representing positive MH—ranging from languishing MH to flourishing MH—and a second continuum ranging from the absence of distress through to the presence of symptoms, or even an MH disorder. Although complementary, these dimensions are distinct [55-57]. The concomitant analysis of these 2 continua provides a more complete description of individuals’ MH status [58,59], particularly in HE [60]. The positive dimension of MH includes psychological well-being, life satisfaction, meaning in life, and recovery, while the negative dimension presents a continuum that includes psychological distress, early warning signs, and psychiatric symptoms [56,61].

In addition, understanding and intervening on student MH requires adopting a developmental approach [62-66]. This approach integrates concepts from several other theoretical models and has the advantage of simultaneously considering the roles of several groups of interrelated factors and their interactions, with the goal of better understanding individual development in relation to the environment [67,68]. Considering these factors is essential, as students’ MH is influenced by social determinants of mental health (SDMH), which include individual, social, environmental, economic, cultural, and political determinants [53]. Some determinants may act as risk factors for developing MH disorders or as aggravating factors in their progression [69,70], while others may act as protective or promotional factors (eg, having a clear and mature identity, having a sense of authenticity and meaning in life, adopting healthy lifestyle habits, benefiting from social support, and evolving in a positive university climate) and prevent the onset of disorders among students [15,71-75]. The OSMHHE recognizes that SDMH must be understood in the light of structural and historical power relations. Thus, the project aims to document the cumulative and differentiated effects of systemic exclusion, stigmatization, racism, sexism, ableism, homophobia, and other forms of discrimination in student trajectories.

In concrete terms, the OSMHHE embodies this conception across all its activities. For example, in its KM efforts, members are working to raise awareness within HEIs about the importance of addressing multiple SDMH. This conception is also reflected in the various themes of the OSMHHE’s 26 axes. Likewise, in its research activities, particular attention is paid to documenting both the positive and negative dimensions of MH and the various SDMH.

##### A Participatory “By-and-for Students” Approach

Anchored in a participatory “by-and-for students” approach, the OSMHHE aims to work in concert with this population by involving them at every stage, from conceptualization to interpretation, and ultimately, to KM [76]. This central role assigned to students helps the OSMHHE move away from the hierarchical relationship that typically exists within HEIs and ensures that student voices remain at the core of the project. Despite inherent challenges (eg, determining the depth of their participation, diminishing the hierarchization of knowledge) [77,78], this approach has the potential to promote better alignment with community needs; coconstruct best practices; and support their adoption, use, and sustainability, as well as share power while fostering capacity and agency of those involved [76,79]. Students can participate across different spheres, including training (eg, with active instruction [80]), educational practices (eg, the *student voice* method [81]), and research, which falls within the broader field of participatory approaches [76,82].

In concrete terms, the OSMHHE was coconstructed with significant contributions from students, including provincial student associations. As a result, this community is actively involved in its governance committees, where it is at the heart of decision-making. To ensure that the perspectives of those most affected by structural inequalities in MH are heard and valued, the OSMHHE ensures that representatives from marginalized groups are included in its governance structures.

Students can take on a number of responsibilities, including participating in discussions and decision-making, helping to plan the activities of each axis, organizing OSMHHE events (eg, conferences, scientific symposia), and evaluating applications for start-up funds that finance OSMHHE flagship projects. In addition, a community of practice is dedicated specifically to student members. Led by 2 student coleaders, it aims to promote the MH and well-being of its members, as well as the development of their knowledge and skills.

##### Governance Based on the Teal Organizational Model

The OSMHHE implements the Teal governance model (as proposed by Laloux [49]), which is characterized by self-governance, holism, and an evolutionary purpose. This type of organizational model has been associated with several benefits, including: (1) higher job satisfaction, attributed to the increased level of autonomy that people enjoy [83]; (2) greater freedom to innovate, which positively affects their engagement [84]; and (3) greater team effectiveness [85]. Such governance has created space for collaborative and shared leadership, both individual and collective, as well as for the embodiment of values that are shared and coconstructed by the active members of the organization [86]. While these findings are encouraging, further studies are needed given their limited number [85,87].

Accordingly, the OSMHHE encourages its axes and their members to exercise leadership that drives its activities forward. For example, suggestions are made to guide their work (eg, annual frequency of meetings, agenda) and update the role of axis leaders. These proposals are intended to support members while allowing them freedom of action. An OSMHHE support team accompanies them, offering assistance with initial setup, agenda preparation, and facilitation. Finally, various mechanisms have been put in place to foster the development of projects (eg, project start-up funds) and the dissemination of results (eg, financial assistance for publication).

##### A KM Infrastructure to Support Change in Culture and Practices

The OSMHHE is defined as a KMI, that is, a set of interconnected components (governance, human and financial resources, KM strategies and activities, processes, etc) that promotes KM by stimulating sharing among various actors (researchers, knowledge users, etc), and facilitates the use of research knowledge to enhance its impact [88-90]. Several KMIs have emerged since the early 2000s [91-93]. These are increasingly recognized as a strategy to support practice improvement and evidence-based decision-making [94-97]. Despite their emergence, very few empirical studies have evaluated them [98]. Current research is more focused on evaluating specific KM activities and initiatives that rely on individuals, such as knowledge brokers, than on assessing KMIs as systems [99-101]. To date, little is known about the roles, governance structures, responsibilities, processes, and activities of KMIs [98]. This is further complicated by the fact that each KMI has unique characteristics that enable it to remain in constant motion as a complex system [102] influenced by a wide range of political, social, organizational, human, and financial factors [92,96,103]. Thus, it is difficult to determine how these KMIs function to produce their impacts [104,105].

A recent evaluation of Canada’s Student Mental Health Network highlights the lack of a standardized evaluation process for assessing this type of KMI [51], despite the acknowledged importance of continuously evaluating these networks’ collaborative efforts to improve partner synergy and ensure effective project functioning [52]. Given this state of knowledge, publications such as this one can contribute to building a body of knowledge that identifies optimal ways to evaluate KMIs. This contribution draws on the KM expertise of several OSMHHE members in evaluating KM strategies or KMI and in supporting the development of professional practices.

Operationally, the OSMHHE KMI includes a support team composed of an OSMHHE coordinator who provides high-level coordination of all activities and supports certain axes, 2 support staff and scientific advisors who assist specific axes and carry out cross-sectoral mandates, a communications specialist, a knowledge broker specializing in KM, an individual responsible for ethical considerations and liaison with the granting agency, as well as students, supporting specific mandates. This team facilitates the work of members and cross-sectoral activities.

In addition, the OSMHHE has a dedicated KM axis whose role has been to help develop an initial KM plan, inspire the axes, evaluate activities, and assess the implementation and impacts of OSMHHE. The initial plan, drawing on knowledge brokers whose work aligns with broad categories of brokering activities [106,107], is ambitious and evolving. For the time being, it includes the following activities: (1) knowledge dissemination and management activities (eg, website, newsletter, member information, *Être et Liberté* podcast by and for students, collection of practice guides, scientific publications), (2) mobilization and networking activities (eg, conferences, colloquia, assemblies); (3) capacity-building activities (eg, interactive workshops or training courses to foster uptake of our guides or productions), and (4) change-support activities (eg, training and support for intermediaries who play a role in supporting HEIs).

### Objectives of the Research Protocol

With the aim of remaining attentive, responsive, and agile with the knowledge it will develop and mobilize in response to the needs of students, institutions, and its members, the OSMHHE has equipped itself with a unique infrastructure and set far-reaching objectives that require both flexible and innovative conceptualization and methodology. The research protocol has three objectives:

1. Develop portraits of student MH and the determinants that support it
2. Evaluate a variety of MH practices
3. Evaluate the implementation and impacts of the OSMHHE as a KMI implementing governance inspired by the Teal organizational model and a participatory approach

## Methods

### Methods for Objective 1: Develop Portraits of Students’ MH

#### Overview

Portraits will be developed based on surveys and complementary research projects. In particular, a first province-wide survey on the MH of students in HE was conducted in Quebec’s HEIs in the fall of 2024 and will be repeated in the fall of 2026.

The specific objectives of these surveys are to:

- Establish a portrait of students’ MH, both positive and negative, in Quebec’s HEIs, and track its evolution over time using the 2 measurement periods
- Generate subpopulation portraits that account for the diversity of individual characteristics and educational trajectories of student populations in Quebec’s HEIs
- Provide each participating institution with portraits of the MH of their student population
- Assess the need for, access to, and use of psychosocial support services within and outside HEIs

#### Creating an Integrated Conceptual Model and Selecting Indicators

To select the dimensions, indicators, and scales to be included in the survey, our team developed a conceptual framework that integrates 3 existing frameworks [47,61,108,109] and conducted a systematic review of the literature specific to the MH of students in HE.

These sources were used to produce a comprehensive portrait of the objects and dimensions to be studied in an MH survey. Inspired by Delphi methodologies [110], they were first subjected to cross-sectional analysis. They were then grouped, merged, or recategorized according to logical criteria to develop a conceptual model specific to our research object, and ultimately, to select the indicators to be included in the survey. This process took into account, among other things, the priorities identified by OSMHHE members.

The selection of variables (scales, questionnaires, items, and questions) assessing the selected indicators was supported by a second literature review focusing on the psychometric qualities of the measurement instruments (validity and reliability), the availability and validated English and French versions as well as the short versions, the royalty-free options, and the number of items.

#### Design of the Provincial Survey on the MH of Students in HE

The survey is based on a cross-sectional, repeated-measures design (2024‐2026). A census-type strategy is used, meaning that the entire Quebec HE student population is surveyed. All 154 post-secondary institutions are targeted, including private, public, and subsidized or nonsubsidized sectors.

To minimize participation biases and obtain satisfactory statistical power, for a population of 415,000 students, a 20% sample size is targeted to give a margin of error of 0.5%, a risk of *α*=5, and statistical power greater than 99.9%. However, a 30% sample would be much preferred, in hopes of achieving better representation of specific populations in line with the OSMHHE population axes. To achieve this rate, several strategies are used to encourage active collaboration from institutions, such as premeetings with representatives of HEIs, collaboration with student associations, and participation incentives through a prize draw.

The survey includes questions on students’ individual characteristics (eg, age, gender identity, sexual orientation, immigration status), MH (eg, positive MH, anxiety symptoms, depressive symptoms, suicidal ideation, and risks), and MH determinants (eg, perception of MH, identity development, authenticity, meaning in life, substance use, screen time, sleep, physical activity). It also covers several systemic contextual variables (eg, social relationships, campus climate, sense of belonging), sexual violence (eg, reporting sexual violence experienced since arriving at the HE institution), and care pathways (eg, help sought from a psychosocial support service, knowledge of psychosocial support services). Examples of survey questions are provided in Multimedia Appendix 1, along with the observational study protocol checklist by Low et al [111] completed for the survey study (Table S1 in Multimedia Appendix 2).

#### Data Collection and Analysis

An invitation to complete an online questionnaire hosted on the Limesurvey platform is sent by email to all students enrolled in HEIs.

At each of the two data-collection periods, descriptive analyses will be carried out to present the results of the various indicators by students’ postsecondary education levels. Inferential analyses will also be conducted to compare the different education levels as well as subpopulations defined by individual characteristics (eg, students with disabilities, immigrant background) and academic contexts (eg, full-time or part-time). Multiple regression and logistic models will be used to target the factors (individual and academic context characteristics, student experience, lifestyle habits) that may predict MH variables. To track changes in MH indicators, repeated-measures analyses of variance will be used to determine whether positive or negative changes have occurred over time. Finally, more complex linear mixed-effects models will be used to determine whether certain determinants explain the changes observed in MH indicators across time.

### Methods for Objective 2: Evaluate MH Practices

#### Overview

The second general objective of the OSMHHE is to evaluate MH practices, programs, and initiatives. More specifically, this involves assessing both their impacts and their implementation and scale-up, as considering these 2 types of impacts often provides clues that could help explain implementation failures or successes [43,45,112,113]. Projects related to this objective will be implemented gradually as promising practices, programs, or initiatives are identified.

#### Evaluation of the Implementation, Sustainability, and Scale-Up of Practices

In line with this objective, the OSMHHE encourages its members to evaluate the implementation, sustainability, and scale-up of promising practices identified. This type of evaluation seeks to highlight the conditions that influence these practices’ adoption, scale-up, and sustainability within HEIs. Targeted initiatives will be prioritized using an analysis tool developed by Ben Charif et al [114], which assesses a practice’s potential for scale-up.

To achieve this objective, the OSMHHE will implement a mixed approach based on the work of several authors, as suggested by Schultes [45] for evaluating the implementation of school-based interventions [115,116]. On the one hand, Proctor et al [115] propose implementation impact analysis as a promising strategy for identifying indicators of success (eg, program accessibility, acceptability, adoption by the community, relevance, feasibility). On the other hand, Damschroder et al [116,117] propose the Consolidated Framework for Implementation Research, which helps identify optimal conditions for implementing promising practices or programs. This mixed approach has already been used by the first author of this paper to evaluate a program aimed at preventing anxiety in young people. Table S1 in Multimedia Appendix 3 provides an overview of the methodological approach adapted for program evaluation in HE.

Although data analysis will vary from project to project, depending on the evaluation context, quantitative descriptive and correlational analyses could be used [118,119]. In addition, drawing on implementation-evaluation tools, path analysis using structural equations may be performed [120]. Finally, thematic analyses could be carried out using qualitative data [121].

#### Evaluation of the Impacts of Different Student MH Practices

The OSMHHE aspires to contribute to the development of a culture of evaluation in HEIs by ensuring that a substantial number of practices are evaluated each year, either independently or with support. The analysis of their impacts will pay specific attention to the differentiated effects associated with membership in historically marginalized groups. The intention is to make sure that interventions do not replicate, or even exacerbate, existing inequalities and that they support true equity in the observed effects. To achieve this ambitious goal, members of the OSMHHE will help support and equip institutions wishing to carry out these evaluations themselves. Initially, a first toolkit will be developed and distributed to guide institutions in their evaluation of training workshops aimed at improving student MH. Adaptations of this toolkit will subsequently be proposed for other types of initiatives (eg, peer support). Support will also be offered to institutions that wish to use the toolkit.

In addition, certain axes—in particular, axis M2 (evaluation of practices to promote MH)—will conduct in-depth evaluations of selected practices based on the following criteria: (1) project scope and cross-sectoral nature, (2) potential project impacts, (3) potential contribution to the advancement of scientific knowledge, (4) nature of the targeted practice (eg, consistency with policy directions, innovation), and (5) presence of conditions likely to facilitate the conduct of the research (eg, level of engagement of the communities proposing the projects).

Data analysis for this component will vary depending on the practices, methodological approach, and tools selected for the evaluation by the institutions. In general, quantitative descriptive, correlational, and comparative analyses [119] could be conducted. In addition, content analyses [76,122] or thematic analyses [121] could be carried out with qualitative data. Table S1 in Multimedia Appendix 4 provides a concrete example of this methodological approach, which is currently being used to evaluate a program aimed at developing the psychosocial skills of students in HE.

### Methods for Objective 3: Evaluate the OSMHHE Implementation and Impacts

#### Overview

The third objective is to evaluate the implementation and impacts of OSMHHE as a KMI that operated with governance inspired by the Teal organizational model and a participatory approach that fosters student mobilization. The intention is to document the innovative nature of the OSMHHE structure by evaluating the development, functioning, and progress toward achieving its objectives. It will be based on the framework of Ziam et al [123] for assessing KM strategies. This framework identifies four components to be considered: (1) the internal context, (2) the external context, (3) the implementation process, and (4) the short-term and medium-term impacts. This flexible framework targets the components and indicators that relate to the conditions for implementation and the impacts of the KM strategy.

#### Design for the Evaluation of the OSMHHE Implementation and Impacts

The design proposed for this evaluation is the case study [124], that is, an in-depth investigation of a specific phenomenon (the case) within its natural context (real-life setting). It will take the form of an embedded case study, with each axis, committee, and work team constituting a unit of analysis [124]. It will rely on data triangulation [76,124] using different sources of information. To avoid overburdening members, data collection will be aligned with the OSMHHE’s regular activities, including accountability reporting.

#### Evaluative Approach

The evaluative approach used is grounded in the principles [125] of developmental evaluation [126], which aims to assess social innovations embedded in complex, dynamic environments. In this approach, the evaluator is involved in the program and seeks “to support the development of complex and dynamic interventions in real time” (p. 2) [127]. This creates strong coherence between the evaluative approach and the establishment of the OSMHHE, whose structure is innovative and whose actions are guided by a shared set of values. This approach is also consistent with the proposed design, as it encourages feedback loops in the development of the infrastructure.

#### Data Collection and Analysis

A convergent mixed methods design will be used, combining qualitative (eg, individual and group interviews, documents, observations) and quantitative data (eg, questionnaires) [128]. Qualitative and quantitative data will be collected and analyzed separately, and the results will then be compared and interpreted jointly.

Approximately 15 semistructured individual interviews [129,130] will be conducted with key members of the OSMHHE, including codirectors, coordinator, support team members, and axis leaders and members until theoretical saturation is reached. Three semistructured group interviews [2] will also be conducted with other OSMHHE members. Depending on the timing of the interviews, the protocols will address topics such as participants’ understanding and perceptions of the OSMHHE’s mission and objectives; strategic planning of actions to achieve these objectives; roles, decision-making processes, and governance; level of engagement and the nature of collaborations; planned or implemented KM strategies; anticipated or observed facilitators and barriers; anticipated or observed outcomes and indicators of success (eg, knowledge development, student training, monitoring of promising practices, etc). All interviews will be recorded and transcribed verbatim.

Qualitative data collection will also include documents already collected by the OSMHHE for operational monitoring (eg, meeting agendas, email newsletters, annual reports, publications). This will involve analyzing the observation notes of the support team members, in line with the participant observation research methodology, where the observer is a recognized participant, in a real-life setting [129,131,132]. Notes will be recorded in a logbook to facilitate systematic documentation. In addition, content analysis will be carried out from discussions on the Teams platform used by the student community of practice to evaluate both its implementation and impacts. Public documents generated by the OSMHHE (eg, meeting minutes) will also be integrated as information sources, consistent with a factist approach [133].

Regarding quantitative data collection, several questionnaires will be developed by the research team and tailored to the context and realities of the OSMHHE and to the different groups of participants surveyed (researchers, clinicians, students, partner institutions). The questionnaires will include both closed-ended and open-ended questions. The data will be collected from 150 members and partners of the OSMHHE. Participants will be surveyed about their level of engagement and satisfaction, the extent to which they feel the OSMHHE’s values are embodied, the challenges and barriers encountered, and the impact of the OSMHHE’s activities and KM practices (eg, MH awareness, improved knowledge on promising practices, changes in practices within HE institutions, implementation of actions, and perceived cultural change in HE).

The qualitative data [122] will be analyzed using content analysis. The data will be transferred to qualitative analysis software (NVivo 11; QSR International) to facilitate data management, coding, and extraction [134]. Quantitative data will be analyzed using IBM SPSS software [119] to generate descriptive statistics. Comparative and correlational analyses will also be conducted to assess the influence of certain variables and to identify trends, including analyses based on sociodemographic factors and correlations between variables related to students’ MH.

### Ethical Considerations

Regarding the provincial survey on the MH of students in HE, ethical approval was obtained from 2 designated research ethics committees (Université de Sherbrooke [2023-3736/Lane] and Cégep de Jonquière [CER-2324-30]). Approval was also secured from all participating institutions, in accordance with institutional requirements and prevailing ethical standards. Although the survey is deemed to be of minimal risk, several ethical considerations are carefully addressed. For example, participation is contingent upon informed consent. As participants create a code for anonymous identification before answering the questionnaire, their participation is completely anonymous, as are their data. Participants were entirely free to choose whether or not to answer the different questions in the survey. A button allowing them to withdraw from the study and not submit their responses was visible and available on every page of the survey. However, because the data were anonymous, it was not possible to withdraw participants’ responses after they had completed and submitted the survey. MH support resources are embedded throughout the questionnaire, and a comprehensive list of available services is provided at both the beginning and the end of the survey. To promote inclusivity and representativeness of the Quebec student population, participants may complete the questionnaire in either French or English. In addition, the principles of equity, diversity, and inclusion are considered and integrated into all documents. This strategy provides nonprobability sampling with voluntary participation. Finally, two CAD $500 (US $353.08) gift cards and eight CAD $100 (US $70.62) gift cards were drawn among all the students who participated in the survey.

## Results

This research project is funded from February 2023 to February 2028. The first of the 2 OSMHHE periodic provincial surveys took place in November 2024. The data were collected from 77 HEIs and 32,212 students. Analyses are currently underway, and the final report was released in November 2025. This survey will be used to produce detailed portraits of the MH status of students in HE, in terms of well-being (positive MH) and of MH difficulties. Identifying MH determinants through this questionnaire will guide HEIs in implementing practices and measures. The survey will contribute to determining whether certain populations are more vulnerable to MH issues and to developing tailored winning strategies for intervention and even prevention. It will also enable a more effective response to the diverse needs of the student population and ensure equitable access to available services.

The 2 survey waves will make it possible to assess changes in the student MH portrait. While maintaining confidentiality, specific reports could be provided to participating HEIs, or to student and professional associations concerned with the issues affecting specific populations (eg, students with disabilities, student athletes). The use of standardized questionnaires, the projected sample size, and the large number of variables identified in the integrated conceptual framework will support the development of a body of scientific knowledge unprecedented in Quebec.

Moreover, around 20 student MH practices are currently being evaluated in terms of their impacts and the conditions under which they are implemented, sustained, and scaled up. Evaluation reports will be published beginning in 2026. These impact evaluations will contribute to understanding their effectiveness and help identify promising practices. Exploring implementation conditions will deepen our understanding of the factors involved in implementing and adopting these practices, and foster their sustainability and scale-up. These findings can also be incorporated into the KM plan. Overall, these evaluations are expected to promote student MH and advance scientific knowledge in this field. A further aim is that the projects will foster change in the culture of practice evaluation within HEIs.

Finally, evaluating the implementation of the OSMHHE infrastructure will help advance knowledge about this type of infrastructure and its functioning. It will also enable feedback loops consistent with the principles-based evaluation approach. In other words, the results generated from the various data collections will allow for continuous monitoring of OSMHHE and its activities, which is essential to the infrastructure’s agility, optimize its operation, and promote its impact. This evaluation will also help advance knowledge on the benefits such infrastructures can bring, including concrete cultural and practice changes in both research (culture of collaboration, interdisciplinarity, and KM) and practice, notably the MH of HE students. It is hoped that this initiative will inspire the development of similar infrastructures elsewhere in the world to foster this cultural change for the benefit of students’ MH.

## Discussion

### Principal Findings

This protocol describes the development of the OSMHHE, a KMI designed to generate, mobilize, and apply scientific and practical knowledge to improve student MH in HE. It offers a unique infrastructure that combines a research mission, aimed at advancing knowledge, with a KM mission focused on supporting cultural and practice change within HEIs. In a context where institutions worldwide are facing an intensification of MH problems among students and increasing calls for systemic approaches, the OSMHHE integrates large-scale data collection, province-wide surveillance, implementation evaluation, assessment of practices, participatory student engagement, and KM within a single coordinated infrastructure. In this regard, it contributes to emerging provincial, national, and international efforts to operationalize learning health principles in HE settings.

First, the objective of developing scientific knowledge through research projects and 2 provincial surveys on student MH and promising practices is consistent with ongoing studies emphasizing the importance of continuing research in this area using rigorous methodologies [135]. These surveys will make a significant contribution to the understanding of student MH by simultaneously considering the positive and negative dimensions of MH and by exploring the explanatory factors that may influence it, including individual, social, and environmental determinants. Repeated data collection may also allow for the monitoring of trends and changes over time and the identification of evolving needs. Because these data will be collected across all HEIs in Quebec, it will be possible to assess the effects of policy orientations, such as the Quebec government’s 2021‐2026 Action Plan on Student Mental Health in Higher Education [47]. Our findings may inform policymakers concerned with student MH in HE. Providing HEIs with personalized reports will enable them to adjust their actions to better support the MH of their student populations. In doing so, those institutions embody the learning health system approach, as proposed by Broglia and Barkham [135] for HE.

Our studies will also help identify the most effective practices (eg, programs, initiatives), in line with our objective of seeking out and monitoring promising practices in MH promotion, awareness, prevention, and intervention in Quebec and internationally. These contributions directly address concerns raised by several authors regarding the difficulty of deciding which are the most effective practices for MH among HE students [26-32].

Second, the objective of supporting the training and mobilizing students involved in MH, with the aim of developing positive leaders, relies fundamentally on our participatory “by-and-for students” approach. The OSMHHE adopts a participatory approach that involves them at every stage: design, interpretation, and dissemination of the results [76]. By prioritizing coconstruction, this approach helps to align actions with real needs, promote knowledge uptake, build capacity, and support genuine empowerment. It is agreed that it is important to involve those most directly concerned—in this case, students—in initiatives, such as the OSMHHE. Indeed, some studies emphasize the importance of involving key members of the target community [2,136,137]. In the KM field, there is shared recognition of the value of interactive approaches that engage stakeholders in various phases, such as knowledge production, adaptation, and dissemination [138]. Partnership and coconstruction are also common features of many KMIs [92,93]. In addition, in terms of the sustainability of actions in academic environments, Herlitz et al [43] emphasize the importance of tailoring interventions to students’ needs and that their involvement is a key factor in the implementation and sustainability of initiatives. The participatory approach adopted by the OSMHHE, therefore, has the potential to improve alignment with local needs, support the coconstruction and uptake of knowledge, and foster capacity-building and empowerment among those involved [76,79]. However, as previously noted, participatory approaches also present a number of challenges [77,78]. To support full student participation in governance, research, and KM actions, the OSMHHE has set up a student community of practice. This initiative fosters their commitment while helping to train the next generation of researchers and leaders in the field of MH.

Third, our goal of fostering KM through the implementation of activities to support cultural and practice changes implies a collective awareness of the importance of such changes. It is widely acknowledged that implementing practices aimed at improving student MH entails substantial cultural change, especially in academic environments [38-41]. Contributing to this transformation requires more than producing research findings: their application must also be promoted. Institutions need support to innovate, implement, scale up, evaluate, and sustain these practices. Considering that HEIs have limited data on promising MH practices and the optimal conditions for their implementation, adoption, sustainability, and scale-up [44,45], OSMHHE studies and KM plans can meaningfully contribute to this needed cultural and practice change.

Finally, it is important to identify the strengths and limitations of this research program. Its major strengths are the scope of the proposed research program and the mobilization around it, the systemic approach, and the participatory “by-and-for students” approach, which enable action across multiple levels, thereby increasing the likelihood of achieving meaningful results. The main limitation is that the current funding is insufficient to fully implement this large-scale research program. Several OSMHHE researchers are seeking (and some have obtained) additional funding to support the projects.

### Conclusions

The OSMHHE’s mission—advancing knowledge and supporting KM to promote and maintain a culture conducive to student MH in HE—is ambitious and driven by its 350 members and collaborators across Quebec and internationally. Thanks to FRQ funding, the OSMHHE has 5 years to accomplish this mission. The coming years will allow us to assess whether the extensive efforts invested in establishing this infrastructure and its activities will bear fruit and generate the intended impacts. The hope is that these impacts will extend well beyond scientific outputs and will contribute to improving student MH and fostering a culture supportive of student MH in HE in Quebec and elsewhere for the benefit of the communities in which these students learn, live, and work.

## Supplementary material

10.2196/83225 Multimedia Appendix 1 Examples of questions in the provincial survey on the mental health of students in higher education.

10.2196/83225 Multimedia Appendix 2 Observational study protocol checklist.

10.2196/83225 Multimedia Appendix 3 Example of a methodological approach to evaluate program implementation, sustainability, and scale-up.

10.2196/83225 Multimedia Appendix 4 Example of a methodological approach to evaluate the zenith program aimed at developing the psychosocial skills of students in higher education.

10.2196/83225 Peer Review Report 1 Peer-review report from the Fonds de recherche du Québec.

## Abbreviations

FRQ: Fonds de recherche du Québec
HE: higher education
HEI: higher education institution
KM: knowledge mobilization
KMI: knowledge mobilization infrastructure
MH: mental health
OSMHHE: Observatory on Student Mental Health in Higher Education
SDMH: social determinants of mental health
