# Supplementary material for: Establishment, Implementation, and Impacts of the Observatory on Student Mental Health in Higher Education in Quebec, Canada: Protocol for a Mixed Methods Research Program
Source: JMIR Res Protoc. 2026 Apr 22;15:e83225. doi: 10.2196/83225 (PMC13102287; doi:10.2196/83225)
Supplement: Multimedia Appendix 1 [file resprot-v15-e83225-s001.pdf]

Examples of Questions in the Provincial Survey on the Mental Health of Students in Higher Education

Individual characteristics

1. What is your age?

2. What term do you use to describe your gender identity?
    a. Female
    b. Male
    c. Transfemale/trans woman
    d. Transmale/trans man
    e. Non-binary
    f. Agender
    g. Queer gender
    h. Gender fluid
    i. Two-spirit
    j. Questioning
    k. I define my gender identity differently, namely:
    l. I do not wish to answer
    m. I do not know

3. What is your immigration status? Select only one answer.
    a. Canadian citizenship by birth
    b. Canadian citizenship by naturalization (immigrants who obtained Canadian citizenship under the Citizenship Act)
    c. Permanent residence
    d. Temporary residence (including international students)
    e. Recognized refugee
    f. No status
    g. Other, please specify:

Mental health

1. Anxiety symptoms (GAD-7) [1]

Over the past 14 days, how often have you been bothered by the following problems?

	0<br>Never	1<br>Several days	2	3

			More than half of the time	Almost every day
Feeling nervous, anxious or on edge.				
Not being able to stop or control worrying.				
Worrying too much about different things.				
Trouble relaxing.				
Being so restless that it is hard to sit still.				
Becoming easily annoyed or irritable.				
Feeling afraid as if something awful might happen.				

2. Depressive symptoms (PHQ-9) [2]

Over the past 14 days, how often have you been bothered by the following problems?

	0<br>Never	1<br>Several days	2<br>More than half of the time	3<br>Almost every day
Little interest or pleasure in doing things.				
Feeling down, depressed, or hopeless.				
Trouble falling or staying asleep, or sleeping too much.				
Feeling tired or having little energy.				
Poor appetite or overeating.				

Feeling bad about yourself – or that you are a failure or have let yourself or your family down.				
Trouble concentrating on things, such as reading the newspaper or watching television.				
Moving or speaking so slowly that other people could have noticed? Or the opposite – being so fidgety or restless that you have been moving around a lot more than usual.				
Thoughts that you would be better off dead or of hurting yourself in some way.				

Mental health determinants

1. In general, would you say your mental health is...?
    a. Excellent
    b. Good
    c. Neither good nor bad
    d. Poor
    e. Very poor

2. Over the past 7 days, how many days did you engage in vigorous physical activity such as carrying heavy loads, mountain biking, or playing hockey/football for at least 10 minutes at a time?
    a. ____ days per week
    b. I did not engage in any vigorous physical activity (skip to question D.3)

3. Currently, what is your total average nightly sleep duration? Do not include the time during which you are awake.
    a. ____ hour(s) ____ minute(s)

Systemic contexts

1. Campus climate

The following questions concern how you feel about your institution since the start of the term (autumn 2024).

	1 Strongly disagree	2 Disagree	3 Somewhat disagree	4 Somewhat agree	5 Agree	6 Strongly agree	7 Not applicable or Do not wish to respond
At my institution, the administration is attentive to students' needs in terms of well-being and mental health.							
My institution offers opportunities to participate in social or community activities on campus.							
My institution offers opportunities to participate in							

cultural, recreational and group leisure activities on campus.							
My institution offers opportunities to participate in political or activist group activities on campus.							
My institution offers opportunities to participate in sports activities on campus.							
My institution offers opportunities to participate in collective religious or spiritual activities on campus.							
There are spaces that allow me to connect with nature on my campus.							
During the day, I feel safe on my campus.							

In the evening, I feel safe on my campus.							
At present, my relationships with other students at my institution are harmonious.							
At present, my relationships with other students at my institution are rewarding.							
At present, my relationships with other students at my institution are satisfactory.							

Sexual violence

Since you started at your higher education institution (college, CEGEP, university, etc.), have you experienced any of the following behaviours from someone affiliated with the same institution? This questionnaire will be given to 1 in 3 respondents at random.

	Yes	No
Section 1		
Being stared at or undressed with the eyes in a way that made you feel uncomfortable.		
Being repeatedly told offensive sexual stories or jokes.		

Being subjected to derogatory remarks about your appearance, body or sexual activities.		
Having someone try to start a conversation with you about sex, even if it was unpleasant (for example, trying to discuss your sex life).		
Being whistled at or called out in a sexual manner.<br>Having someone make offensive or embarrassing gestures towards you with sexual connotations.		
Making insulting or hurtful comments with sexual connotations.		
Being exposed naked or scantily clad in a way that made you feel uncomfortable or embarrassed.		
Asking intrusive or persistent questions about sexuality or the body.		
Questions asked only if the participant has reported experiencing any of the behaviours listed in this section.		
Has this happened at least once in the last 12 months?		
Was the person or persons who committed any of the above behaviours in a position of power over you (did they have authority over you)?		

Care pathway

1. Since the start of the term, have you felt that you needed help to overcome a situation related to your mental or emotional health?
    a. Yes
    b. No

1.1. Have you sought help from the psychosocial support service (psychologist, social worker, etc.)
at your educational institution?

a) Yes, but I was unable to get help.

b) Yes, and I got help.
c) No

1.2. Who did you consult?
a) Psychiatrist
b) Psychologist
c) Nurse
d) Social worker
e) Counsellor
f) Other(s), please specify: __________

This is a Multimedia Appendix to a full manuscript published in the J Med Internet Res. For full copyright and citation information see http://dx.doi.org/10.2196/jmir.83225

References:

1. Spitzer RL, Kroenke K, Williams JBW, Löwe B. A brief measure for assessing generalized anxiety disorder: The GAD-7. Arch Intern Med. 2006;166(10):1092-1097. doi:10.1001/archinte.166.10.1092
2. Kroenke K, Spitzer RL, Williams JBW. The PHQ-9. J Gen Intern Med. 2001;16(9):606-613. doi:10.1046/j.1525-1497.2001.016009606.x
