# Supplementary material for: Establishment, Implementation, and Impacts of the Observatory on Student Mental Health in Higher Education in Quebec, Canada: Protocol for a Mixed Methods Research Program
Source: JMIR Res Protoc. 2026 Apr 22;15:e83225. doi: 10.2196/83225 (PMC13102287; doi:10.2196/83225)
Supplement: Multimedia Appendix 2 [file resprot-v15-e83225-s002.pdf]

Observational Study Protocol Checklist

Table S1. Observational study protocol checklist [1]

Item	Item no.	Explanation	Page number in the study protocol
Title	1	Quebec National Survey on the Mental Health of Higher Education Students and its Determinants	NA
Introduction<br>Background/rationale	2 and 3	**Introduction**<br>Faced with the growing challenges related to student mental health (SMH) in Quebec, the Ministry of Higher Education (MES) launched the Action Plan on Student Mental Health in Higher Education – 2021-2026 in 2021 [2]. The aim is to support college and university networks in implementing practices that promote well-being, flourishing mental health (MH) and the development of the full potential of students in higher education (SHE).<br><br>Among the measures is the creation of the Observatory on Student Mental Health in Higher Education (OSMHHE), funded by the Fonds de recherche du Québec (FRQ, 2023-2028). Co-directed by professors Julie Lane (University of Sherbrooke) and Benjamin Gallais (University of Quebec in Chicoutimi), the OSMHHE' mission is to contribute to the advancement and mobilisation of knowledge to promote and maintain a culture conducive to student mental health (SME) in higher education.<br><br>OSMHHE has four main objectives: a) To develop scientific knowledge through research and investigation into SMÉ and promising practices in SM; b) To contribute to the training and mobilisation of PÉES in relation to SM in order to make them positive leaders; c) Monitor and track promising practices in SM promotion, awareness, prevention and intervention; and d) Promote knowledge mobilisation by implementing several activities to support this vast change	4-5

in culture and practices. OSMHHE has nearly 350 members (professors, researchers, students, practitioners, etc.) divided into five mission areas (including the area 'Assessment of student mental health and its determinants,' which plays a central advisory role in relation to this survey), seven population areas and 14 thematic areas.

As part of its first objective, OSMHHE launched its first provincial survey of all higher education institutions in Quebec in November 2024. The second survey will take place in 2026.

The main objectives are:

1. To establish a portrait of the mental health (positive mental health and symptoms of anxiety and depression) of SHE and monitor its evolution over time;
2. To generate sub-population portraits that take into account the diversity of individual characteristics and academic trajectories of SHE;
3. To assess the need for, access to, and use of psychosocial support services within and outside educational institutions.

The secondary objectives are:

1. To examine the links between MS and the academic context;
2. To examine the links between MS and the student experience on campus;
3. To examine the links between MS and its behavioural and health determinants.

This report presents the findings of this survey, placing them in their scientific and methodological context. The first section briefly outlines the theoretical foundations that guided the design of the survey, as well as the main methodological choices. The results are then presented in an integrated manner and interpreted in order to develop recommendations to support SHE in higher education settings.

Context

The transition to adulthood for students in higher education

The transition to adulthood is a pivotal stage, characterised both by the need to adapt and by multiple opportunities for development. It requires many adjustments related to acquiring new roles, greater autonomy and financial independence. For many young adults, this

period coincides with the start of higher education, whether at CEGEP, college or university, which accentuates the challenges and issues of adaptation.

At the same time, this stage represents a period of opportunity and consolidation. The maturation of the brain promotes better planning, self-regulation and management of risky behaviours [3]. Cognitive skills become more sophisticated, particularly abstract thinking and complex reasoning, facilitating adaptation to the demands of adult life [4]. This period is also conducive to the development of quality social relationships [5], which can act as protective factors that promote well-being [5,6].

The period between the ages of 18 and 29 is also marked by identity exploration and crucial decisions about educational, professional and personal paths [7]. Together, these processes support the identification of a direction that gives meaning to life, promotes motivation and well-being among SHE [8].

This developmental period is therefore accompanied by increased vulnerability to MS. In fact, nearly half of all MS disorders emerge before the age of 18, and more than 60% before the age of 25. International studies conducted on very large samples of young adults indicate that 31% to 38% of them were at risk of developing an SM disorder in the past 12 months [9,10]. These disorders are accompanied by symptoms that can affect cognitive function, psychosocial adjustment, motivation, satisfaction and academic performance [11,12,13], while increasing the risk of social isolation, school dropout and suicidal behaviour [12,13,14,15].

On the other hand, this same period also represents a strategic window of opportunity for promoting mental health among SHE [16]. Supporting these young adults during a major transition in their lives not only helps prevent the development of mental health issues but also promotes well-being and cultivates resilience [17,18].

Student mental health in Canada

In Canada, SMH is a major concern. A large-scale survey [19] conducted among a broad sample of more than 11,000 post-secondary students from 16 institutions of higher education reveals that 33% of post-secondary students experience severe psychological distress, 38% experience high levels of stress, 59% are at risk of isolation, and 36% are at risk of suicide. In addition,

nearly 3% reported having made at least one suicide attempt in the past year. For its part, the study by King et al. indicates that 33% of SHE have clinically significant anxiety symptoms and 28% have clinically significant depressive symptoms [20]. Furthermore, 33% report using psychoactive substances (mainly alcohol and cannabis) and 29% report suicidal thoughts [20].

With regard to positive MS, data from two studies indicate that between 24% and 32% of SHE have flourishing MS [19,21]. However, the majority fall into the moderate category, with proportions ranging from 51% to 68%, revealing a level of well-being that is neither languishing nor optimal. Finally, between 9% and 17% of SHE experiences languishing MS, a profile associated with low levels of emotional, psychological and social well-being. Compared to the general Canadian population, where 77% of adults report thriving MS [22], SHE stand out with a significantly higher prevalence of non-thriving MS, including a much higher proportion of people with moderate MS (compared to 22% in the general population) and languishing MH (compared to 1.5%). These differences highlight that the majority of SHE have a lower level of well-being than that observed in the overall Canadian adult population.

Student mental health in Quebec

In Quebec, various studies and surveys have documented the mental health of higher education students. Recently, a survey by Généreux et al. revealed that just over half of SHE report moderate to severe symptoms of anxiety or depression [23]. In the college network, the most recent Provincial Survey of the CEGEP Student Population 2 (SPEC-2), conducted among thousands of SHE from 35 Cégeps, found that 81% of them identified stress as a significant challenge [24]. Among the difficulties explored in relation to MS, anxiety is the most frequently reported, affecting 43% of SHE, while depression is mentioned in a smaller proportion, 29% [24]. Similarly, in terms of positive MS, the results of a recent study of college students show that approximately 40% of college students have flourishing MS, while 50% have moderate MS and nearly one in ten have languishing MS, indicating fragile levels of emotional, psychological and social well-being [25].

These issues have been around for a long time. In fact, a 2017 study of a sample of more than 12,000 higher education students from eight Cégeps revealed that more than a third of them experienced very high levels of anxiety [26]. In universities, a Quebec-wide survey conducted by the Quebec Student Union (QSU) showed that 58% of SHE experienced high levels of

		psychological distress, and that one in five experienced depressive symptoms requiring medical or psychological support [27]. Similarly, the survey conducted by the *Fédération des associations étudiantes du campus de l'Université de Montréal* of just over 10,000 university students indicated that 54% had felt the need or considered consulting professional mental health services in the past 12 months, mainly for stress or anxiety problems (in 80% of cases), followed by exhaustion, depression or low mood (in 69% of cases) [28]. Surveys conducted by the QSU and the FAÉCUM also reported suicidal ideation in 8% and 9% of SHE, respectively, and suicide attempts in approximately 1% of them [27,29]. Finally, according to Moissac et al., between 3% and 14% of PÉES presented with languishing MH, while 26% to 55% presented with flourishing MH [30]. These results suggest that the mental health difficulties observed after the pandemic are not a new phenomenon, but rather a continuation of worrying trends that were already well established among students in higher education institutions. This perspective provides a better understanding of recent developments in mental health, particularly in light of the changes observed in recent years.<br><br>*Changes in mental health over time and with the pandemic*<br>Based on trends observed over time, various studies converge to indicate a marked increase in MH issues among SHE, both internationally [31,32] and in Canada [33]. For example, the study by Linden et al. highlights a significant increase in stress, mental health disorders and suicidal behaviour among the student population [33].<br><br>This trend could be explained by a number of factors, including, for example, destigmatisation campaigns that encourage greater recognition of psychological difficulties and promote awareness (which could lead to an increase in the frequency of MH disorders observed in studies), as well as the increasing complexity and severity of these conditions, or various barriers to accessing care, such as the perception of a lack of resources and limited knowledge of available services [34,35,36,37]. The COVID-19 pandemic has undoubtedly had a negative impact on MH, as confirmed by several international studies [38,39,40], in Canada [41,42] and in Quebec [43,44,45,46]. In this context, longitudinal monitoring of SMH appears essential, both to document changing needs and to inform prevention, promotion and service organisation initiatives in higher education settings.<br><br>*Limitations of previous studies/surveys*	

		Despite the wealth of existing data, several methodological limitations hinder a comprehensive understanding of SMH in Quebec [47]. Some studies recruited participants via social media, which may introduce bias in terms of representativeness. Others did not use standardised questionnaires, limiting the comparability and rigour of the results. Few Canadian or Quebec studies have adopted a repeated measures approach to track the evolution of MH over time and identify risk and protective factors. Furthermore, no study has simultaneously evaluated college and university students using identical measurement instruments, allowing for comparisons between SHE, in order to identify the factors that promote better SMH according to the level of education. Finally, few surveys have led to concrete recommendations and changes in practices at all levels of the system.	
Objectives	4	The main objectives are:<br>1. To establish a portrait of the mental health (positive mental health and symptoms of anxiety and depression) of SHE and monitor its evolution over time;<br>2. To generate sub-population portraits that take into account the diversity of individual characteristics and academic trajectories of SHE;<br>3. To assess the need for, access to, and use of psychosocial support services within and outside educational institutions.<br><br>The secondary objectives are:<br>1. To examine the links between MS and the academic context;<br>2. To examine the links between MS and the student experience on campus;<br>3. To examine the links between MS and its behavioural and health determinants.	12
Method<br>Study design<br><br>Setting	5 and 6	The survey will adopt a repeated cross-sectional design with two waves of data collection (or phases 1 and 2) to account for the instability of the post-secondary student population (entries into higher education, dropouts or breaks, transitions between levels of education, etc.). Two separate data collection phases, the first in November 2024 and the second scheduled for November 2026, will be conducted among all students enrolled in participating higher education institutions (public and subsidised private). Each participant will receive a unique identification code, generated from certain responses, allowing the data of those who responded to both phases to be matched. Although each collection is cross-sectional in nature, the use of an identical questionnaire for both phases makes it possible to conduct a longitudinal analysis for the subsample responding to both waves. Phase 1 of the survey was distributed in	13-14

		the form of an online questionnaire, designed with LimeSurvey© and sent to students by email via their institution.	
Participants	9	The survey follows a census-type approach, meaning that all students enrolled in higher education institutions are invited to take part. As a result, the study is based on non-probability sampling, combining voluntary and network sampling [48].	13-14
Variables<br><br>Data sources/measurement	12 and 13	1. Individual characteristics<br>a. Age<br>b. Level of education<br>c. Educational institution in Quebec<br>d. Field of study<br>e. Year of programme<br>f. Which Quebec university as place of study<br>g. Level of study<br>h. Study status<br>i. Proportion of course time devoted to studies<br>j. Distance learning<br>k. Proportion of distance learning<br>l. Difficulty with academic success<br>m. Sex assigned at birth<br>n. Term used to describe gender identity<br>o. Frequency of feeling discriminated against because of gender identity on campus<br>p. Term that describes sexual orientation<br>q. Frequency of feeling discriminated against because of sexual orientation on campus<br>r. Ethnicity<br>s. Frequency of feeling discriminated against because of ethnicity on campus<br>t. Immigration status<br>u. Place of birth<br>v. Do you have two mothers or two fathers?<br>w. Place of birth of your parents<br>x. Highest level of education completed by your parents<br>y. Are your parents deceased? If so, how old were you at the time of their death?	13-14

		z. Current relationship status or marital status<br>aa. Mother tongue<br>bb. Are you a student-athlete recognised by your institution?<br>cc. Sports groups<br>dd. Sport practised and recognised by your institution<br>ee. Have you consulted a mental performance consultant in the last six months?<br>ff. Disability status<br>gg. Impairments that may affect daily activities (e.g., studies, social life, household chores)<br>hh. Disclosure of disability status to the educational institution<br>ii. Category or categories of disability status or diagnosis received<br>jj. Have you received a diagnosis from a recognized health professional (psychologist, doctor, neuropsychologist, speech therapist, etc.)?<br>kk. Use of accommodation/adjustment measures, support or assistance services<br>ll. Classification of limitations in daily life<br>mm. Frequency of perceived discrimination due to disability<br>nn. Person who has received youth protection services<br>oo. Postcode of main place of residence<br>pp. Current place of residence (e.g., university residence, off campus, etc.)<br>qq. Dependent children<br>rr. Gross personal income<br>ss. Main source of income<br>tt. Perception of having enough money to meet one's needs<br>uu. Employment or business ownership<br>vv. Average number of hours worked per week<br>ww. Employment related to studies or desired career<br>xx. Stress associated with workdays<br><br>2. Mental health status<br>a. Positive mental health (The Mental Health Continuum-Short Form) [49]<br>b. Mental well-being (Warwick-Edinburg Mental Well-being Scale) [50]<br>c. Perceived stress (Perceived Stress Scale) [51]<br>d. Anxiety symptoms (General Anxiety Disorder-7) [52]	

		e. Depressive symptoms (Patient Health Questionnaire-9) [53]<br>f. Suicidal ideation and risks (Suicidal ideation attributes scale) [54]<br>g. Satisfaction regarding life<br><br>3. Mental health determinants<br>a. Perception of one's general health<br>b. Perception of one’s physical health<br>c. Perception of one’s mental health<br>d. Self-esteem (Rosenberg Self-Esteem Scale) [55]<br>e. Substance use (Alcohol, Smoking and Substance Involvement Screening Test (ASSIST), version 3.0) [56]<br>f. Substance comportments and impacts<br>g. Playing games of chance and gambling<br>h. Screen time<br>i. Competence<br>j. Mental health literacy<br>k. Questions on psychosocial support inside and outside the institution (e.g., feel the need to get help, the professional consulted, satisfaction regarding the help received, knowledge of campus services and outside services)<br>l. Emotional wellbeing strategies<br><br>4. Physical activity<br>a. Number of days of intense physical activities<br>b. Number of minutes per day of intense physical activities<br>c. Number of days of moderate physical activities<br>d. Number of minutes per day of moderate physical activities<br>e. Number of days of walking<br>f. Number of minutes of walking<br>g. Number of days spent walking<br>h. Number of minutes spent walking<br>i. Number of days spent sited<br>j. Number of minutes spent sited	

		5. Sleep<br>a. Number of hours of sleep needed to feel rested<br>b. Total time of sleep by night, in general<br>c. Usual times when getting up and going to bed when there are work or classes the following day<br>d. Usual times when getting up and going to bed when there are no work or classes the following day<br>e. Satisfaction regarding sleep<br>f. To what extent do sleep difficulties disrupt daily functioning<br><br>Systemic contexts<br>1. Social support (Social Provisions Scale-10) [57]<br>2. Solitude<br>3. Campus climate<br>4. Sense of belonging<br>5. Eco-anxiety<br>6. Impact of academic factors on mental health<br>7. Authenticity<br>8. Identity (Dimensions of Identity Development Scale) [58]<br>9. Sens of life (Meaning of Life Questionnaire) [59]<br><br>Sexual violence<br>1. Experience of sexual behaviours from a person affiliated with the same institution	
Bias	14	Following approval from two designated review committees (Université de Sherbrooke and Cégep de Jonquière) and thanks to a personalised approach to the ethics committees of each institution, ethical approval was obtained for all participating institutions, in accordance with institutional requirements and current standards. The survey, deemed to be of minimal risk, is completely anonymous and requires informed consent. Support resources are integrated throughout the questionnaire, and a complete list of services is available at the beginning and end of the survey.	13, 16, 17

		To minimise participation bias and obtain sufficient statistical power, we are counting on high participation rates. To this end, we are employing several strategies:<br>1) Active collaboration with institutions: a) Preliminary meetings with numerous representatives of post-secondary institutions have been and will continue to be held to ensure that they feel involved in the survey process, and b) Individual profiles for each institution will be provided at each data collection stage;<br>2) Collaboration with student representatives (Fédération étudiante collégiale du Québec; Quebec Student Union), who will mobilise their local associations in the field to disseminate and promote the surveys (e.g., reminders on Facebook sites, organisation of events to promote mental health on campuses during the data collection period, including promotion of the survey, etc.);<br>3) Incentives through a random draw (3 iPad-type tablets per collection) to motivate student participation, even among those who do not feel concerned by mental health issues, which is a participation bias often observed in census-type surveys.<br><br>The survey will be available in French and English to ensure that it is as inclusive and representative as possible. In addition, emphasis will be placed on the principles of equity, diversity and inclusion in the production of the online survey (e.g., information form and survey questions accessible to blind and visually impaired persons – text-to-speech reading); the general principles of accessibility of the Web Accessibility Initiative will be taken into account. Also, to minimise the burden on participants, the total duration of the survey will be 20 to 30 minutes maximum. The survey will be pre-tested with students before its launch. This pre-test will be conducted in two stages:<br>1) Cognitive interview-type pre-test (assessment of the comprehensibility and acceptability of the questions by a representative panel of students);<br>2) Pre-test under 'real' conditions (assessment of completion time).	
Study size	15	For a population of 415,000 students, samples of 20%, 25% and 30% would give us a margin of error of 0.5%, a risk α = 5 and a power greater than 99.9%. However, a 30% sample would be much preferred, in hopes of achieving better representation of specific populations in line with the OSMHHE population axes.	13

Quantitative variables<br>Statistical methods	16 and 17	The statistical analyses performed using SPSS [60,61] will mainly be based on descriptive and comparative analyses. In terms of description, the socio-demographic profile, mental health indicators, and results on perceptions of the campus climate, care pathways, population profiles, and lifestyle profiles will be presented using relative frequencies (%). Where relevant, measures of central tendency and dispersion, such as the mean and standard deviation, will be used. In terms of comparisons, cross-tabulation tables and graphs will be presented to provide a qualitative comparison of the descriptive results.	14
	16 and 18	More in-depth analyses, including statistical comparison analyses, regression models, multivariate analyses, and longitudinal analyses between collection phases, will be carried out. These analyses will allow for a more detailed examination of the factors associated with SMH and will enable us to track its evolution over time.	14
	16 and 19	It should be noted that there will be missing data for all the survey questions. As such, the results will sometimes be accompanied by details on the sample size (n) according to sociodemographic characteristics, to better contextualise the scope of the percentages presented. It should be noted that to avoid constraining respondents, no questions are going to be mandatory. In addition, some questions will be administered conditionally or randomly, which will explain why the associated sample size may occasionally be smaller than the total sample.	N/A
	16 and22	More in-depth analyses, including statistical comparison analyses, regression models, multivariate analyses, and longitudinal analyses between collection phases, will be carried out. These analyses will allow for a more detailed examination of the factors associated with SMH and will enable us to track its evolution over time.	14

This is a Multimedia Appendix to a full manuscript published in the J Med Internet Res. For full copyright and citation information see http://dx.doi.org/10.2196/jmir.83225

References:

1. Low GK, Subedi S, Omosumwen OF, et al. Development and validation of observational and qualitative study protocol reporting checklists for novice researchers (ObsQual checklist). Eval Program Plann. 2024;106:102468. doi:10.1016/j.evalprogplan.2024.102468

2. Ministère de l'Enseignement supérieur. Plan d'action sur la santé mentale étudiante en enseignement supérieur 2021-2026. Gouvernement du Québec; 2021. URL: https://cdn-contenu.quebec.ca/cdn-contenu/adm/min/education/publications-adm/enseignement-superieur/PASME.pdf [accessed 2025-01-25]
3. Taber-Thomas B, Pérez-Edgar K. Emerging adulthood brain development. In: Arnett JJ, ed. The oxford handbook of emerging adulthood. 1st ed. Oxford University Press; 2016:126-141. doi:10.1093/oxfordhb/9780199795574.013.15
4. King PM, Kitchener KS. Cognitive development in the emerging adult: The emergence of complex cognitive skills. In: Arnett JJ, ed. The oxford handbook of emerging adulthood. 1st ed. Oxford University Press; 2016:105-125. doi: 10.1093/oxfordhb/9780199795574.013.14
5. Padilla-Walker LM, Memmott-Elison MK, Nelson, LJ. Positive relationships as an indicator of flourishing during emerging adulthood. In: Padilla-Walker LM, Nelson LJ, eds. Flourishing in emerging adulthood: Positive development during the third decade of life. Oxford University Press; 2017:212-236.
6. Gómez-López M, Viejo C, Ortega-Ruiz R. Well-being and romantic relationships: A systematic review in adolescence and emerging adulthood. Int J Environ Res Public Health. 2019;16(13):2415. doi:10.3390/ijerph16132415
7. Arnett JJ. A longer road to adulthood. In: Arnett, JJ, ed. Emerging adulthood: The winding road from the late teens through the twenties. 3rd ed. Oxford University Press; 2023:1-26.
8. Bronk KC, Baumsteiger R. The role of purpose among emerging adults. In: Padilla-Walker LM, Nelson LJ, eds. Flourishing in emerging adulthood: Positive development during the third decade of life. Oxford University Press; 2017:45-66. doi:10.1093/acprof:oso/9780190260637.003.0004
9. Auerbach RP, Mortier P, Bruffaerts R, et al. The WHO world mental health surveys international college student project: Prevalence and distribution of mental disorders. J Abnorm Psychol. 2018;127(7):623-638. doi:10.1037/abn0000362
10. Auerbach RP, Mortier P, Bruffaerts R, et al. Mental disorder comorbidity and suicidal thoughts and behaviors in the world health organization world mental health surveys international college student initiative. Int J Methods Psych Res. 2019;28(2):e1752. doi:10.1002/mpr.1752
11. Duffy A, Keown-Stoneman C, Goodday S, et al. Predictors of mental health and academic outcomes in first-year university students: Identifying prevention and early-intervention targets. BJPsych Open. 2020;6(3):e46. doi:10.1192/bjo.2020.24

12. Lipson SK, Eisenberg D. Mental health and academic attitudes and expectations in university populations: Results from the Healthy Minds Study. J Ment Health. 2018;27(3):205-213. doi:10.1080/09638237.2017.1417567
13. Markoulakis R, Kirsh B. Difficulties for university students with mental health problems: A critical interpretive synthesis. RHE. 2013;37(1):77-100. doi:https://doi.org/10.1353/rhe.2013.0073
14. Gili M, Castellví P, Vives M, et al. Mental disorders as risk factors for suicidal behavior in young people: A meta-analysis and systematic review of longitudinal studies. J Affect Disord. 2019;245:152-162. doi:10.1016/j.jad.2018.10.115
15. Tong Y, Wang S, Cao L, et al. School dropouts related to mental disorders: A systematic review and meta-analysis. Asian J Psychiatr. 2023;85:103622. doi:10.1016/j.ajp.2023.103622
16. Martínez-García A, Valverde-Montesino S, García-García M. Promoting mental health in higher education: Towards a model of well-being factors in emerging adulthood. Int J Qual Stud Health Well-being. 2024;19(1):2408831. doi:10.1080/17482631.2024.2408831
17. McGorry P, Mei C, Dalal N, et al. The Lancet Psychiatry Commission on youth mental health. Lancet Psychiatry. 2024;11(9):731-774. doi:10.1016/S2215-0366(24)00163-9
18. O'Connor M, Casey L. The Mental Health Literacy Scale (MHLS): A new scale-based measure of mental health literacy. Psychiatry Res. 2015;229(1-2):511-516. doi:10.1016/j.psychres.2015.05.064
19. American College Health Association. American college health association-national college health assessment III: Reference group executive summary Fall 2022. 2023. Accessed January 19, 2026. https://www.acha.org/wp-content/uploads/2024/07/NCHA-III_FALL_2022_REFERENCE_GROUP_EXECUTIVE_SUMMARY.pdf
20. King N, Pickett W, McNevin SH, et al. Mental health need of students at entry to university: Baseline findings from the U-Flourish Student Well-Being and Academic Success Study. Early Interv Psychiatry. 2021;15(2):286-295. doi:10.1111/eip.12939
21. Peter T, Roberts LW, Dengate J. Flourishing in life: An empirical test of the dual continua model of mental health and mental illness among Canadian university students. Int J Ment Health Promotion. 2011;13(1):13-22. doi:10.1080/14623730.2011.9715646
22. Gilmour H. Positive mental health and mental illness. Health Rep. 2014;25(9):3-9.

23. Généreux M, Bergeron J, Bourassa L, Brisson Sylvestre M-P, Fafard, A-C, Melançon M-È. Enquête sur la santé psychologique des 12-25 ans - Rapport 2023. 2023. Accessed January 19, 2026. https://reussirestrie.ca/wp-content/uploads/2023/05/RAPPORT_mai_2023_vf.pdf
24. Gaudreault MM., Richard É, Charron M, Tardif S, Gallais B, Gaudreault M. Enquête sur la réussite à l'enseignement collégial, à partir des données du SPEC 2 2022 Expérience étudiante, motivation, santé mentale et réussite à la deuxième session d'études. 2024. Accessed January 19, 2026. https://ecobes.cegepjonquiere.ca/media/tinymce/Enquete_ReussiteCollegial/EnqueteReussite_SyntheseSPEC2_2024-06-27.pdf
25. Surprenant R, Bezeau D, Tiraboschi GA, et al. Associations between youth lifestyle habits, sociodemographic characteristics, and health status with positive mental health: A gender-based analysis in a sample of Canadian postsecondary students. Prev Med Rep. 2025;51:103015. doi:10.1016/j.pmedr.2025.103015
26. Gosselin M-A, Ducharme R. Détresse et anxiété chez les étudiants du collégial et recours aux services d'aide socioaffectifs. Service soc. 2017;63(1):92-104. doi:10.7202/1040048ar
27. Bérard J, Bouchard J, Roberge, V, LeBel P, Allard S, Lessard F-É. Enquête « Sous ta façade »: Enquête panquébécoise sur la santé psychologique étudiante, Automne 2018. 2019. Accessed January 19, 2026. https://unionetudiante.ca/Media/publicDocuments/Rapport-enquete-sous-ta-facade.pdf
28. Chénard Poirier L-A, St-Gelais A, Forest S, Bérard J, LeBel P, Bazinet K. Enquête sur l'utilisation étudiante des services de santé psychologique à l'Université de Montréal. 2017. Accessed January 19, 2026. https://www.faecum.qc.ca/ressources/documentation/avis-memoires-recherches-et-positions-1/enquete-sur-l-utilisation-etudiante-des-services-de-sante-psychologique-a-l-universite-de-montreal
29. Lessard F-É, St-Gelais A, LeBel P, Vanier A-C. Enquête sur la santé psychologique étudiante. 2016. Accessed January 19, 2026. https://www.faecum.qc.ca/ressources/documentation/avis-memoires-recherches-et-positions-1/enquete-sur-la-sante-psychologique-etudiante
30. de Moissac D, Graham JM, Prada K, Gueye NR, Rocque R. Mental health status and help-seeking strategies of Canadian international students. CJHE. 2020;50(4):52-71. doi:10.47678/cjhe.vi0.188815

31. Duffy ME, Twenge JM, Joiner TE. Trends in mood and anxiety symptoms and suicide-related outcomes among U.S. undergraduates, 2007–2018: Evidence from two national surveys. J Adolesc Health. 2019;65(5):590-598. doi:10.1016/j.jadohealth.2019.04.033
32. Oswalt SB, Lederer AM, Chestnut-Steich K, Day C, Halbritter A, Ortiz D. Trends in college students' mental health diagnoses and utilization of services, 2009-2015. J Am Coll Health. 2020;68(1):41-51. doi:10.1080/07448481.2018.1515748
33. Linden B, Boyes R, Stuart H. Cross-sectional trend analysis of the NCHA II survey data on Canadian post-secondary student mental health and wellbeing from 2013 to 2019. BMC Public Health. 2021;21(1):590. doi:10.1186/s12889-021-10622-1
34. Côté JE, ed. Youth development in identity societies: Paradoxes of purpose. Routledge; 2019.
35. Moghimi E, Stephenson C, Gutierrez G, et al. Mental health challenges, treatment experiences, and care needs of post-secondary students: A cross-sectional mixed-methods study. BMC Public Health. 2023;23(1):655. doi:10.1186/s12889-023-15452-x
36. Wiens K, Bhattarai A, Pedram P, et al. A growing need for youth mental health services in Canada: Examining trends in youth mental health from 2011 to 2018. Epidemiol Psychiatr Sci. 2020;29:e115. doi:10.1017/S2045796020000281
37. Wiens K, Bhattarai A, Dores A, et al. Mental health among Canadian postsecondary students: A mental health crisis? Can J Psychiatry. 2020;65(1):30-35. doi:10.1177/0706743719874178
38. Buizza C, Bazzoli L, Ghilardi A. Changes in college students mental health and lifestyle during the COVID-19 pandemic: A systematic review of longitudinal studies. Adolescent Res Rev. 2022;7(4):537-550. doi:10.1007/s40894-022-00192-7
39. Lee B, Krishan P, Goodwin L, et al. Impact of COVID-19 mitigations on anxiety and depression amongst university students: A systematic review and meta-analysis. J Glob Health. 2023;13:06035. doi:10.7189/jogh.13.06035
40. Lemyre A, Palmer-Cooper E, Messina JP. Wellbeing among university students during the COVID-19 pandemic: A systematic review of longitudinal studies. Public Health. 2023;222:125-133. doi:10.1016/j.puhe.2023.07.001
41. King N, Pickett W, Rivera D, et al. The Impact of the COVID-19 Pandemic on the mental health of first-year undergraduate students studying at a major Canadian university: A successive cohort study. Can J Psychiatry. 2023;68(7):499-509. doi:10.1177/07067437221094549

42. Zhu J, Racine N, Xie EB, et al. Post-secondary student mental health during COVID-19: A meta-analysis. Front Psychiatry. 2021;12:777251. doi:10.3389/fpsyt.2021.777251
43. Veilleux N, Leblanc-Pageau R, Lévesque C, Dupéré V. Rapport de l'enquête nationale « Derrière ton écran » : Une enquête de la FECQ sur les impacts de la COVID-19 sur la condition étudiante au collégial. 2021. Accessed January 19, 2026. https://stationsme.ca/wp-content/uploads/2023/11/Rapport-final-DTE_109eCo_Zoom.pdf
44. Gallais B, Blackburn M-È, Gaudreault M. Après deux ans de pandémie : comment vont nos étudiant.e.s du collégial sur les plans psychologique et scolaire? Communication presented at: Colloque du Réseau des sciences humaines des collèges du Québec. 2022.
45. Tremblay L, Brunette M. Ensuring the success and engagement of university students in times of pandemic: The issue of mental health. DRHJ. 2022;5(1):1-14. doi:10.28984/drhj.v5i2.351
46. Union étudiante du Québec. Enquête courte sur la santé psychologique étudiante au Québec en temps de pandémie de la COVID-19. 2021. Accessed January 19, 2026.
47. Lane J, Therriault D, Dupuis A, et al. The Impact of the COVID-19 pandemic on the anxiety of adolescents in Québec. Child Youth Care Forum. 2022;51(4):811-833. doi:10.1007/s10566-021-09655-9
48. Fortin M-F, Gagnon J. Fondements et étapes du processus de recherche : méthodes quantitatives et qualitatives. 4th ed. Chenelière éducation; 2022.
49. Keyes CLM, Wissing M, Potgieter JP, et al. Evaluation of the Mental Health Continuum-Short Form (MHC-SF) in Setswana-speaking South Africans. Clin Psychol Psychother. 2008;15(3):181-192. doi:10.1002/cpp.572
50. Tennant R, Hiller L, Fishwick R, et al. The Warwick-Edinburgh Mental Well-being Scale (WEMWBS): Development and UK validation. Health Qual Life Outcomes. 2007;5(1):63. doi:10.1186/1477-7525-5-63
51. Cohen S, Kamarck T, Mermelstein R. A global measure of perceived stress. J Health Soc Behav. 1983;24(4):385-396. doi:10.2307/2136404
52. Spitzer RL, Kroenke K, Williams JBW, Löwe B. A brief measure for assessing generalized anxiety disorder: The GAD-7. Arch Intern Med. 2006;166(10):1092-1097. doi:10.1001/archinte.166.10.1092
53. Kroenke K, Spitzer RL, Williams JB. The PHQ-9: Validity of a brief depression severity measure. J Gen Intern Med. 2001;16(9):606-613. doi:10.1046/j.1525-1497.2001.016009606.x

54. van Spijker BAJ, Batterham PJ, Calear AL, et al. The Suicidal Ideation Attributes Scale (SIDAS): Community-based validation study of a new scale for the measurement of suicidal ideation. Suicide Life Threat Behav. 2014;44(4):408-419. doi:10.1111/sltb.12084
55. Rosenberg M. Society and the Adolescent Self-Image. Princeton, NJ: Princeton University Press; 1965.
56. Humeniuk R., Henry-Edwards S, Ali, RL, et al. The Alcohol, Smoking and Substance Involvement Screening Test (ASSIST): Manual for use in primary care. 2010. Accessed January 19, 2026. https://iris.who.int/server/api/core/bitstreams/d38b0238-268a-495f-8139-4b69bb12b7ad/content
57. Cutrona C, Russell D, Rose J. Social support and adaptation to stress by the elderly. Psychol Aging. 1986;1(1):47-54. doi:10.1037//0882-7974.1.1.47
58. Mastrotheodoros S, Motti-Stefanidi F. Dimensions of Identity Development Scale (DIDS): A test of longitudinal measurement invariance in Greek adolescents. Eur J Dev Psychol. 2017;14(5):605-617. doi:10.1080/17405629.2016.1241175
59. Steger MF, Frazier P, Oishi S, Kaler M. The Meaning in Life Questionnaire: Assessing the presence of and search for meaning in life. J Couns Psychol. 2006;53(1):80-93. doi:10.1037/0022-0167.53.1.80
60. Field AP. Discovering statistics using IBM SPSS Statistics: And sex and drugs and rock ‘n’ roll. 4th ed. Palgrave Macmillan; 2013.
61. *IBM SPSS Statistics for Windows.* Version 29. IBM Corp. 2023. Accessed November 6, 2025. https://www.ibm.com/products/spss-statistics
