# Supplementary material for: Establishment, Implementation, and Impacts of the Observatory on Student Mental Health in Higher Education in Quebec, Canada: Protocol for a Mixed Methods Research Program
Source: JMIR Res Protoc. 2026 Apr 22;15:e83225. doi: 10.2196/83225 (PMC13102287; doi:10.2196/83225)
Supplement: Multimedia Appendix 3 [file resprot-v15-e83225-s003.pdf]

Example of a Methodological Approach to Evaluate Program Implementation, Sustainability, and Scale-Up

Table S1.

Implementation Dimensions [1]	Research Questions [1]	Research Questions [2]	Data Collection Tools
**Acceptability**	What is the level of satisfaction among stakeholders regarding the different components of the program?	What factors influence acceptability?	▪ Student surveys<br>▪ Facilitators' of the program logbooks<br>▪ Focus group interviews with the program implementation committee<br>▪ Acceptability of Implementation Measure [3]
**Adoption**	Which institutions decided to implement the program and why? What is the level of adoption?	What factors influence adoption?	▪ Focus group interviews with the program implementation committee
**Appropriateness**	What are staff perceptions regarding the compatibility of the program with students' needs?	What factors influence appropriateness?	▪ Focus group interviews with the program implementation committee<br>▪ Implementation Appropriateness Measure [3] and SUBSIT measure for the sustainability of behaviour support interventions at school [4] questionnaires completed by the

			program implementation committee<br>▪ Facilitators' of the program logbooks
**Feasibility**	Is the program applicable to the institution's context?	What factors influence feasibility?	▪ Focus group interviews with the program implementation committee<br>▪ SUBSIT measure for the sustainability of behaviour support interventions at school [4] and Feasibility of Implementation Measure [4] questionnaires completed by the program implementation committee<br>▪ Facilitators' of the program logbooks
**Cost**	What are the implementation costs?	What factors influence implementation costs?	▪ Administrative data<br>▪ Identification of key program components and their costs
**Fidelity**	Is the program delivered as intended by the development team? Have modifications been made?	What factors influence fidelity?	▪ Focus group interviews with the program implementation committee<br>▪ Facilitators' of the program logbooks<br>▪ Observation of 10% of program sessions

**Scale-Up**	What is the level of scale-up?	What factors influence scale-up?	▪ Administrative data<br>▪ Focus group interviews with the program implementation committee
**Sustainment / Sustainability**	Do institutions continue the program in subsequent years?	What conditions influence sustainability?	▪ Administrative data<br>▪ SUBSIT measure for the sustainability of behaviour support interventions at school [4] questionnaire completed by the program implementation committee

This is a Multimedia Appendix to a full manuscript published in the J Med Internet Res. For full copyright and citation information see http://dx.doi.org/10.2196/jmir.83225

References:

1. Proctor E, Silmere H, Raghavan R, Hovmand P, Aarons G, Bunger A, et al. Outcomes for implementation research: conceptual distinctions, measurement challenges, and research agenda. Adm Policy Ment Health. 2011;38(2):65-76. doi:10.1007/s10488-010-0319-7
2. Damschroder LJ, Aron DC, Keith RE, Kirsh SR, Alexander JA, Lowery JC. Fostering implementation of health services research findings into practice: a consolidated framework for advancing implementation science. Implement Sci. 2009;4:50. doi:10.1186/1748-5908-4-50
3. Weiner BJ, Lewis CC, Stanick C, Powell BJ, Dorsey CN, Clary AS, et al. Psychometric assessment of three newly developed implementation outcome measures. Implement Sci. 2017;12(1):108. doi:10.1186/s13012-017-0635-3
4. McIntosh K, MacKay LD, Hume AE, Doolittle J, Vincent CG, Horner RH, et al. Development and initial validation of a measure to assess factors related to sustainability of school-wide positive behaviour support. J Posit Behav Interv. 2011;13(4):208-218. doi:10.1177/1098300710385348
