# Supplementary material for: Establishment, Implementation, and Impacts of the Observatory on Student Mental Health in Higher Education in Quebec, Canada: Protocol for a Mixed Methods Research Program
Source: JMIR Res Protoc. 2026 Apr 22;15:e83225. doi: 10.2196/83225 (PMC13102287; doi:10.2196/83225)
Supplement: Multimedia Appendix 4 [file resprot-v15-e83225-s004.pdf]

Example of a Methodological Approach to Evaluate Zenith Program Aimed at Developing the Psychosocial Skills of Students in Higher Education

The data are collected online from participants at three different points in time: before (T1), after (T2), and 4 months after (T3) the intervention.

Table S1.

Program objective	Data Collection Tools
To evaluate the effects of participation in the program on the development of certain knowledge and psychosocial skills among students.	▪ A questionnaire is used to document changes in knowledge and skills and the factors that influence them [1]. Students who participate in the workshop rate their perceived level of knowledge and skills mastery on a scale of 1 to 10 before (T1) and after (T2) the program. At T2, they must also identify what they learned during the program. Students' willingness to change will also be assessed at T2 and T3 (need to change, willingness to change, ability to change, and perceived relevance of the knowledge and skills targeted by the program).
To evaluate the program's effects on various dimensions, such as psychological well-being, perceived stress, cognitive flexibility, emotional regulation, sense of social self-efficacy, and emotional skills.	▪ Standardized questionnaires with proven psychometric properties will be completed online by participants at three measurement points (T1, T2 and T3) : Scale for measuring manifestations of psychological well-being [2]; Perceived Stress in a probability sample [3]; Multidimensional Psychological Flexibility Inventory [4]; Emotion Regulation Skills Questionnaire [5]; Self-efficacy Questionnaire [6]; Emotional Skills Profile [7].

This is a Multimedia Appendix to a full manuscript published in the J Med Internet Res. For full copyright and citation information see
http://dx.doi.org/10.2196/jmir.83225

References:

1. Lauzier M, Denis D. Accroître le transfert des apprentissages: Vers de nouvelles connaissances, pratiques et expériences. Québec, QC: Presses de l'Université du Québec; 2016.
2. Massé R, Poulin C, Dassa C, Lambert J, Bélair S, Battaglini MA. Élaboration et validation d'un outil de mesure du bien-être psychologique: L'ÉMMBEP. Can J Public Health. 1998;89(5):352-357. doi:10.1007/BF03404490
3. Cohen S, Kamarck T, Mermelstein R. A global measure of perceived stress. J Health Soc Behav. 1983;24(4):385-396. doi:10.2307/2136404
4. Rolffs JL, Rogge RD, Wilson KG. Disentangling components of flexibility via the Hexaflex Model: Development and validation of the multidimensional psychological flexibility inventory (MPFI). Assessment. 2018;25(4):458-482. doi:10.1177/1073191116645905
5. Grant M, Salsman NL, Berking M. The assessment of successful emotion regulation skills use: Development and validation of an English version of the Emotion Regulation Skills Questionnaire. PLoS One. 2018;13(10):e0205095. doi:10.1371/journal.pone.0205095
6. Muris P. A brief questionnaire for measuring self-efficacy in youths. J Psychopathol Behav. 2001;23(3):145-149. doi:10.1023/A:1010961119608
7. Brasseur S, Grégoire J, Bourdu R, Mikolajczak M. The Profile of Emotional Competence (PEC): Development and validation of a self-reported measure that fits dimensions of emotional competence theory. PLoS One. 2013;8(5):e62635. doi:10.1371/journal.pone.0062635
