# Supplementary material for: Establishment, Implementation, and Impacts of the Observatory on Student Mental Health in Higher Education in Quebec, Canada: Protocol for a Mixed Methods Research Program
Source: JMIR Res Protoc. 2026 Apr 22;15:e83225. doi: 10.2196/83225 (PMC13102287; doi:10.2196/83225)
Supplement: Peer Review Report 1 [file resprot-v15-e83225-s005.pdf]

Rapport d'évaluation consensus final de la demande

Programme Observatoire sur la santé mentale étudiante en enseignement supérieur

RÉFÉRENCES DE LA DEMANDE / REFERENCES OF THE PROPOSAL

N° de la demande	324014
Chercheur Principal/Chercheuse Principale<br>Cocandidat/Cocandidate	Julie Lane
Établissement	Université de Sherbrooke
Cocandidat/Cocandidate	Benjamin Gallais
Établissement	Cégep de Jonquière
Titre de la demande	Observatoire sur le bien-être et la santé mentale étudiante en enseignement supérieur : pour des connaissances au service de l'action

RAPPORT D'ÉVALUATION CONSENSUS
**Commentaires du comité scientifique**

Section 1: **Constitution de l'équipe (20 %)**

Forces :

- Direction conjointe entre l'université (directrice) et le cégep (codirecteur) ce qui soutient la diversité des institutions.
- Grande diversité des disciplines, autant parmi les chercheurs/chercheuses et les collaborateurs/collaboratrices.
    - Les sciences du sport, les sciences de la santé communautaire, la psychologie, les sciences de la psychoéducation et de la pédagogie, les relations industrielles, la médecine familiale, la psychiatrie, les relations interculturelles, le travail social, les sciences infirmières, la réadaptation et la sociologie
- Exhaustivité des disciplines représentées, notamment parmi les professions qui ont trait aux soins en santé mentale, est une des forces de la demande
- Représentation à peu près égale entre les universités et les cégeps
- Appui important des milieux universitaires, collégiaux et des associations étudiantes ce qui permet d'avoir une bonne représentation géographique
- Effort d'impliquer une multitude d'acteurs sur le terrain et solide ancrage dans le milieu étudiant
- La liste des collaborateurs comprend plusieurs fédérations et organisations étudiantes
- Effort pour impliquer une multitude d'acteurs sur le terrain qui démontre un souci d'ancrer le projet dans le tissu universitaire
- Une personne de la population étudiante et un membre du corps professoral codirigent chaque axe thématique. Les rôles de chacun sont clairement décrits. Ce binôme étudiant-chercheur est un des points forts de la demande.
- Un modèle de participation collaborative est mentionné qui insiste sur l'influence égalitaire que chacun peut exercer dans le processus de décision
- Les étudiants et les étudiantes sont inclus à chaque niveau de la gouvernance.
- Les décideurs locaux participent au comité d'orientation stratégique
- Les directeurs de projets ont de l'expérience sur la recherche en appui aux politiques, sur la mise en œuvre et sur les grandes études longitudinales.
- Il y a un aperçu clair de chaque axe et des personnes qui seront impliquées
- Un grand nombre de besoins spécifiques de la population étudiante est identifié

Points à améliorer :

- La grande diversité des disciplines peut être difficile à superviser et à gérer, mais comme chaque équipe thématique travaille de façon autonome avec une structure de communication

et de gestion transversales pour assurer des liens entre les thèmes et augmenter la faisabilité du projet.

Section 2: **Excellence de la programmation (35 %)**

Forces :

- La proposition s'articule autour de la structure des soins par étapes
- Le projet met l'accent non seulement sur la collecte des données, mais aussi sur la mise en œuvre des résultats de la recherche et leur utilisation immédiate.
- Axe thématique sur les structures institutionnelles et politiques qui indique un souci de répondre aux besoins futurs.
- Importance accordée à la déstigmatisation qui est souvent un frein au recours aux soins
- L'axe des autosoins est une piste prometteuse et très pertinente
- Enquête longitudinale en deux temps, ce qui répond aux attentes formulées dans l'appel de demande.
- Cocréation des projets, des interventions et des communications en collaboration avec les étudiants de façon à répondre à leurs besoins
- L'articulation entre les chercheurs, les chercheuses, les étudiants, les étudiantes et les professionnels de la santé est très prometteuse
- Importance mise sur l'implication des étudiantes et des étudiants au projet avec des binômes étudiants chercheurs
- Rôle actif des étudiants et des étudiantes non seulement dans les tâches de recherche dans un souci de formation, mais aussi leur propre prise en charge
- L'approche décrite pour chacun de ces objectifs est étayée par l'utilisation de modèles validés et clarifiée dans plusieurs exemples
- La gouvernance fondée sur la confiance et la bienveillance avec des mesures concrètes de mise en œuvre de ces valeurs, ce qui crée une atmosphère constructive.
- Lien clair entre les axes et les mesures du PASME

Points à améliorer :

- Le comité se demande si la subvention de démarrage de 5 000 $ est-elle suffisante pour que les projets puissent être mis en œuvre puis évalués.
- La façon dont la stigmatisation sera abordée pourrait être mieux décrite
- Le choix de 26 axes thématiques peut être trop grand pour assurer une gestion équitable afin d'aller au bout des objectifs de chaque axe
- Moins d'axes auraient permis une meilleure description de chacun
- Les quatre derniers axes sont des copier-coller des objectifs du programme

Recommandation :

- Une condensation du nombre d'axes pourrait faciliter la faisabilité de chacun.

Section 3 : **Enquête longitudinale (20 %)**

Forces :

- Conception et approche théorique claires
- Tableau de bord qui permet des analyses secondaires
- Prise en compte des aspects de la santé mentale positive et des facteurs protecteurs dans le milieu étudiant (p. ex., l'équilibre entre les aspects de la vie) qui sont moins étudiés en général

Points à améliorer :

- Pas de mention de la taille de l'échantillon
- On s'attend à un taux de participation élevé, ce qui ne se présente pas dans les enquêtes de ce type internationales. Même avec le fort engagement des associations étudiantes, il faudrait clarifier s'il est possible d'avoir plus de 20% de taux de réponse et quels moyens seront pris pour augmenter le taux de réponse.
- Les outils utilisés pour caractériser la santé mentale et notamment les problèmes de santé mentale s'intéressent seulement aux problèmes récents (symptômes de dépressions, d'anxiété, comportements suicidaires, détresse psychologique)
- Les outils, notamment ceux qui mesurent les aspects positifs de la santé mentale sont-ils adéquats ou adaptés au contexte universitaire? Ce n'est pas précisé dans la demande.
- Si 30% des étudiants répondent et que 40 % de ceux-ci présentent un symptôme quelconque. Ce sera difficile d'arriver à un nombre suffisant pour tirer des conclusions solides.
- Le choix d'une méthode d'échantillonnage non probabiliste aurait pu être mieux justifié
- Le choix des deux temps de mesure aurait pu aussi être mieux expliqué
- Quelles sont les personnes qui représenteront les établissements ? Comment l'équipe prévoit-elle minimiser les liens de dépendance possible entre les représentants des établissements et les participantes et les participants ? Y a-t-il un risque que les personnes étudiantes se sentent obligées de participer à l'enquête par crainte de subir des conséquences négatives ?
- La création d'une base de données accessible à plusieurs personnes comprenant les résultats de l'enquête et des données démographiques pourrait soulever de défis quant à l'anonymat. Il sera donc important dans la présentation des résultats de mettre en place des mesures pour éviter l'identification des personnes participantes. Quelles mesures seront prises pour minimiser ces risques ?

Recommandation

- Le projet devrait s'intéresser si possible à la psychopathologie tout au long de la vie. Souvent les problèmes apparaissent avant les études supérieures. La santé mentale préalable a un effet sur l'état de santé mentale pendant les études supérieures et cela pourrait enrichir l'étude.
- Les points à améliorer sont nombreux mais il serait facile d'y remédier. Ils sont mentionnés dans un souci d'améliorer l'enquête longitudinale.

Section 4: **Mobilisation des connaissances (20 %)**

Forces :

- Description très détaillée et complète de publics cibles et des retombées attendues
- Mise en place une infrastructure distincte pour mobiliser les connaissances
- Nombreuses collaborations avec les partenaires qui pourront aider à la diffusion des résultats
- Modèle de formation pour les formateurs
- Présence dans le consortium d'experts reconnus et identifiés au niveau national, ce qui pourrait faciliter la mise en œuvre des recommandations qui découleront des travaux de l'Observatoire.

Points à améliorer :

- Rien à signaler

Section 5: **Équité, diversité et inclusion (EDI) (5 %)**

Forces :

- Bonne prise en compte de ces aspects
- Souci d'inclure des personnes en situation de handicap et des personnes issues de l'immigration ou d'autres origines culturelles
- Les axes thématiques montrent en effet une diversité de populations étudiantes avec leurs besoins spécifiques
- Les activités de formation en EDI
- Le projet a impliqué deux centres de recherche pour réaliser spécifiquement l'inclusion des personnes en situation de handicap et des personnes issues de l'immigration ou d'autres origines culturelles. Ces deux centres ont pour mission d'influencer toutes les autres activités et partenaires afin d'assurer l'équité, la diversité et l'inclusion.

Points à améliorer

- Rien à signaler

NOTE GLOBALE FINALE / FINAL GLOBAL SCORE	
Cote Finale	83.25
Rang	1
Nombre de demandes évaluées	2

Ce rapport fait état du récapitulatif des commentaires et impressions du comité scientifique conjoint à la suite des discussions en comité plénier. Les commentaires reflètent la note finale donnée au projet pour un classement final.
